# Tartrate-resistant acid phosphatase (TRAP/*ACP5*) promotes metastasis-related properties via TGFβ2/TβR and CD44 in MDA-MB-231 breast cancer cells

**DOI:** 10.1186/s12885-017-3616-7

**Published:** 2017-09-15

**Authors:** Anja Reithmeier, Elena Panizza, Michael Krumpel, Lukas M. Orre, Rui M. M. Branca, Janne Lehtiö, Barbro Ek-Rylander, Göran Andersson

**Affiliations:** 1 Karolinska Institutet, Department of Laboratory Medicine (LABMED), H5, Division of Pathology, F46, Karolinska University Hospital, Huddinge, 141 86 Stockholm, Sweden; 2 Karolinska Institutet, Department of Oncology-Pathology (OnkPat), K7, Research Group Janne Lehtiö, Box 1031, 171 21 Solna, Sweden

**Keywords:** *ACP5*, TRAP, 5-PNA, TGFβ2, CD44, MDA-MB-231

## Abstract

**Background:**

Tartrate-resistant acid phosphatase (TRAP/*ACP5*), a metalloenzyme that is characteristic for its expression in activated osteoclasts and in macrophages, has recently gained considerable focus as a driver of metastasis and was associated with clinically relevant parameters of cancer progression and cancer aggressiveness.

**Methods:**

MDA-MB-231 breast cancer cells with different TRAP expression levels (overexpression and knockdown) were generated and characterized for protein expression and activity levels. Functional cell experiments, such as proliferation, migration and invasion assays were performed as well as global phosphoproteomic and proteomic analysis was conducted to connect molecular perturbations to the phenotypic changes.

**Results:**

We identified an association between metastasis-related properties of TRAP-overexpressing MDA-MB-231 breast cancer cells and a TRAP-dependent regulation of Transforming growth factor (TGFβ) pathway proteins and Cluster of differentiation 44 (CD44). Overexpression of TRAP increased anchorage-independent and anchorage-dependent cell growth and proliferation, induced a more elongated cellular morphology and promoted cell migration and invasion. Migration was increased in the presence of the extracellular matrix (ECM) proteins osteopontin and fibronectin and the basement membrane proteins collagen IV and laminin I. TRAP-induced properties were reverted upon shRNA-mediated knockdown of TRAP or treatment with the small molecule TRAP inhibitor 5-PNA. Global phosphoproteomics and proteomics analyses identified possible substrates of TRAP phosphatase activity or signaling intermediates and outlined a TRAP-dependent regulation of proteins involved in cell adhesion and ECM organization. Upregulation of TGFβ isoform 2 (TGFβ2), TGFβ receptor type 1 (TβR1) and Mothers against decapentaplegic homolog 2 (SMAD2), as well as increased intracellular phosphorylation of CD44 were identified upon TRAP perturbation. Functional antibody-mediated blocking and chemical inhibition demonstrated that TRAP-dependent migration and proliferation is regulated via TGFβ2/TβR, whereas proliferation beyond basal levels is regulated through CD44.

**Conclusion:**

Altogether, TRAP promotes metastasis-related cell properties in MDA-MB-231 breast cancer cells via TGFβ2/TβR and CD44, thereby identifying a potential signaling mechanism associated to TRAP action in breast cancer cells.

**Electronic supplementary material:**

The online version of this article doi:(10.1186/s12885-017-3616-7) contains supplementary material, which is available to authorized users.

## Background

Tartrate-resistant acid phosphatase (TRAP/*ACP5*) is a metalloenzyme of the category of acid phosphatases [1] that is synthesized as a monomeric proenzyme (TRAP 5a, 35 kDa) [2]. A disulfide linked heterodimer (TRAP 5b) with an N-terminal fragment of 20–23 kDa joined to the 16 to 17 kDa C-terminal part originates from post-translational cleavage of the monomeric form, which significantly increases phosphatase activity [3]. TRAP derived from different mammalian sources reveals almost identical homology at the amino acid sequence level and identical biochemical properties [4–7]. During bone resorption, TRAP is secreted into the resorption lacuna of active osteoclasts, where it dephosphorylates the bone matrix protein osteopontin (OPN), thereby promoting osteoclast detachment and migration [8]. Additionally, TRAP has been suggested to regulate OPN bioactivity in autoimmune conditions [9–11]. The isoform TRAP 5b was proposed as a serum marker for bone metastases in various types of primary cancers [12–16]. Interestingly, TRAP has also been detected in several cancer cells and tissues (breast, ovarian, cervical cancer and malignant melanoma) and its expression level correlates with the severity of the tumor [17–19]. Moreover, high TRAP expression correlates with reduced tumor- and metastasis-free survival in malignant melanoma [20], and with decreased overall survival and increased incidence of metastasis in hepatocellular cancer [21]. In gastric cancer, elevated TRAP expression is an independent risk factor for peritoneal dissemination and is associated with shorter patient survival [22]. In lung cancer, patients with high TRAP expression had a significantly lower overall survival than the patients with low TRAP expression [23].

Altogether, previous studies underscore the potential clinical relevance of TRAP to monitor cancer development and progression; nevertheless, underlying cellular and molecular processes remain unclear.

TRAP was shown to interact intracellularly with the Transforming growth factor β (TGFβ) receptor interacting protein-1 (TRIP-1), thereby activating TGFβ receptor type II (TβR2) and osteoblast differentiation through the Mothers against decapentaplegic homolog 2/3 (SMAD2/3) pathway at sites of prior bone resorption [24]. Furthermore, TRIP-1 knock-down abrogates osteoblast differentiation and proliferation [25]. TRAP 5a interaction with TRIP-1 has also been demonstrated in mouse pre-adipocytes [26].

TGFβ ligands exist in three highly homologous isoforms, TGFβ1, TGFβ2 and TGFβ3 and are part of a large family of structurally related secreted cytokines [27–29]. Upon ligand binding to the constitutively active serine/threonine kinase TβR2 [30], the latter forms a hetero-oligomeric complex with the type I receptor (TβR1). TβR2 then trans-phosphorylates TβR1, ultimately leading to the transcription of various target genes via both SMAD- and non-SMAD mediated pathways (reviewed in [29, 31–34]). TGFβ acts as tumor repressor in early stages of tumorigenesis and as an oncogene in late stages [28]. For instance, in breast cancer patients expression of TGFβ was increased in tumor tissue [35, 36] and was associated with disease progression [36, 37]. TGFβ2 has been proposed as a predictive marker for breast cancer, as high levels of TGFβ2 correlate with advanced tumor stage and shortened survival [38]. Additionally, TGFβ2 was reported as a catalyzer of TGFβ signaling through an autocrine loop [39]. Finally, TβR1 contain a single cytoplasmic binding site for Cluster of differentiation 44 (CD44) [40], suggesting a potential interaction between the TGFβ pathway and CD44, a cancer-associated glycoprotein previously reported as an OPN receptor [41]. Activated CD44 stimulates the serine/threonine kinase activity of TβR1, which in turns increases SMAD2/3 phosphorylation [40].

Aim of this study was to delineate by proof-of-concept, how TRAP promotes cellular properties related to metastasis in breast cancer cells at advanced state. As there is only limited knowledge about the molecular perturbations and possible substrates of TRAP, global phosphoproteomic and proteomic analysis was applied to connect possible signaling mechanisms to the TRAP-dependent phenotypic changes.

## Methods

### Reagents

**Matrix proteins:** bovine milk OPN, previously purified by our group [42, 43], murine Engelbrecht-holm-swarm Laminin-1 (Lam I, Sigma-Aldrich, #L2020), human plasma Fibronectin (FN, Life Technologies, #PHE0023), human recombinant natural Vitronectin (VN, Life Technologies, #PHE0011), Cultrex® Rat Collagen I, (Col I, Trevigen, Cat# 3440–100-01), human placenta Collagen IV (Col IV, Merck Millipore, Cat# CC076) (10 μg/ml).

**Antibodies:** rabbit antibody serum against total TRAP (raised by immunization of New Zealand rabbits [44], 1:1000, TRAP 5a: 37 kDa, TRAP 5b: 16 and 25 kDa), mouse anti-β-Actin (1:1000, 42 kDa; Cat# 8224, Abcam), rabbit anti-TGFβ2 (Western blotting 1:1000, 48 kDa; ICC 1:200; Cat# 113670, Abcam), secondary donkey anti-mouse (Licor IRDye® 800CW, Cat# 925–32,212; 1:15,000); donkey anti-rabbit (Licor IRDye® 680RD, Cat# 926–68,073; 1:15,000); goat anti-rabbit (Licor IRDye® 680RD Cat# 926–68,071; 1:15,000); secondary goat anti-rabbit Alexa 488 (1:100, Cat#A11008, Life Technologies).

**Recombinant protein, blocking antibodies and inhibitory compounds:** rat monoclonal IgG2A Isotype Control (respective similar concentration, Novus Biologicals, MAB006, 54,447), rat monoclonal anti-CD44 (10 μg/mL, Novus Biologicals, Hermes-1, NBP2–22530), rabbit polyclonal anti-TGFβ2 (0,25 μg/mL, R&D systems, AB-12-NA); TGFβ receptor type I/type II kinases inhibitor LY2109761 (2 μM, Santa Cruz; CAS 700874–71-1, PubChem CID 11655119), Human recombinant TGFβ1 (10 ng/mL, R&D systems, 240-B), TRAP inhibitor 5-PNA (200 μM, 5-phenylnicotinic acid, Maybridge code CC24201, Sigma-Aldrich code CDS013984; PubChem CID 346160, previously characterized by our group [45]).

### Cell transfection and culture

MDA-MB-231 breast cancer cell line was obtained from American Type Culture Collection (Manassas, U.S., ATCC® Number: HTB-26™). MDA-MB-231 were previously stably transfected with the eukaryotic expression vector pcI-neo containing the full size rat TRAP [46] and different subpopulations maintained in complete medium (RPMI 1640, 10% fetal bovine serum, 0.1 mg/mL Gentamicin) (Life technologies, Carlsbad, CA, U.S.) at 37 °C in a 5% CO_2_ humidified atmosphere. Cells were continuously tested for contamination with the MycoAlert™ mycoplasma detection kit (Lonza, Cat# LT07).

Knockdown of rat TRAP was achieved by the use of different custom cloned MISSION shRNA constructs within the lentivirus plasmid vector pLKO.1-puro containing ampicillin and puromycin antibiotic resistance genes (Sigma Aldrich, St. Louis, MO, U.S.). Transfections were done with Escort II transfection reagent according to the manufacturer’s instructions. 1 μg of purified DNA was complexed with 5 μl transfection reagent and applied to the cells for 24 h. Cells transfected with plasmids encoding antibiotic resistance were selected by culture of complete medium supplemented with 1 μg/mL puromycin (Sigma Aldrich, St. Louis, MO, U.S.). TRAP gene expression in the cells bearing a knockdown was quantified according to a detailed description in the Additional file 1: Material and Methods.

### Tartrate-resistant acid phosphatase activity assay

Cell lysates and conditioned media were prepared according to a detailed description in the Additional file 1: Material and Methods. TRAP activity in cell lysate and medium was measured under optimal enzyme conditions using 10 mM p-nitrophenyl-phosphate as a synthetic substrate (Sigma Aldrich) as previously described [45]. Enzymatic activity was calculated in enzyme Units according to Lambert-Beer and normalized to total protein concentration for lysate or per 10^6^ cells per 24 h for medium.

### Immunoblotting

Cell lysates and conditioned media were prepared according to a detailed description in the Additional file 1: Material and Methods. 25–50 μg of total protein and normalized volumes of corresponding medium were subjected to SDS-PAGE (Mini-PROTEAN®TGX™ precast gel, Biorad) and transferred to PVDF membrane (Trans-Blot turbo mini PVDF packs, Biorad) according to the manufacturer’s instructions. Unspecific binding was blocked by 3% bovine serum albumin (BSA) in PBS for 1 h at room temperature (R.T.) and protein bands detected by subsequent incubation with respective primary antibodies in each 3% BSA overnight (o.n.) at 4 °C and fluorescently labelled secondary antibodies for 1 h at R.T.. The membranes were washed with TBST (20 mM Tris- HCl pH 7.5, 500 mM NaCl, 0.05% Tween-20) after antibody incubations and visualized in the Licor Odyssey Fc Imager and quantified by densitometry with the Licor Image Studio software 3.1.4 (Licor Biosciences, Lincoln, NE, U.S.) upon normalization to β-Actin expression for lysates.

### Anchorage-independent growth

6-well plates were precoated with a bottom layer of 0.8% low melting Agarose solution (Sigma Aldrich, A9414) prepared in complete medium and gel formation allowed for at least 1 h at R.T.. After gelling, 10,000 cells were incorporated in a 0.35% Agarose solution and overlaid on the previous high Agarose-containing lower layer. During regular feeding, cells were allowed to form colonies for 3 weeks and agarose gels fixed in formaldehyde (Solvecco). Colonies were stained in 0.01% Crystal violet solution in formaldehyde o.n. and 1X images taken. Images were changed to binaries and threshold adjusted on all images using Image J 1.48 software. Colony number and sizes were quantified by the “count particles” function with a threshold of 50 pixels as minimum detection limit and inclusion of colonies with circularity from 0 to 1.

### Cell growth assay

Fifty thousand cells were seeded in 48-well plates in complete medium and let adhere and grow for 24 h or 48 h. The cells were washed with PBS, fixed in formaldehyde and stained for 5 min in 0.1% Toluidine blue. Excess color was washed away with PBS and color dissolved for 5 min in 50% EtOH/50 mM HCl. Absorbance was measured at 630 nm in a PowerWave HT Microplate Spectrophotometer (Biotek, Winooski, VT, U.S.).

### Cell proliferation

Fifty thousand cells were seeded into glass 8-well chambers (Labtek II, 154534) and let adhere o.n.. After a PBS wash, medium was changed, respectively, conditionally containing blocking antibodies or inhibiting compounds or deficient in serum and maintained for another 24 h. Following, the cells were pulsated with 10 μM EdU for 1 h and subsequently fixed in 4% formaldehyde for 10 min. Fluorescent staining was performed according to the manufacturer’s protocol with the Click-iT® Plus EdU Alexa Fluor 488 Imaging Kit (Cat# C10637, Invitrogen, Life Technologies Europe BV, Stockholm, Sweden). Confocal images were acquired in the Nikon A1+ confocal laser microscope system and image batch analysis performed in the Nis Elements Advance research imaging software 4.1.0 (Nikon). For automated cell counting, thresholding was adapted to experimental controls and nuclei with circularity greater than 0.1 and size (diameter) greater than 5 μm taken into account.

### Cell morphology

Bright field phase contrast images of cells cultured in complete medium were taken at equal confluence and at different time points in a Nikon Eclipse TE300 Inverted microscope equipped with a DS-Fi1 digital microscope camera and a DS-U2 camera control unit (Nikon, Tokyo, Japan). Ratios of cell length to cell width were acquired for all cells using the Straight line tool in Image J 1.48 program. A minimum of 200 cells was randomly measured per time point and ratio medians calculated.

### Live cell wound migration

Wound migration experiments were performed and analyzed as previously described [45]. Medium was respectively enriched with blocking antibodies, chemical inhibitors or compounds.

### Transwell migration assay

Transwell assays membranes (Corning Incorporated Costar-Transwell CLS3422-48EA) were precoated on the lower side of the insert with 10 μg/mL of matrix proteins in PBS under humidified atmosphere at 4 °C for 24 h. Following, 200,000 cells were seeded into the upper chamber of the Transwell and both wells filled with serum-free medium. Migration was allowed for two different time periods during linear increase (OPN: 8 h, 22 h; FN: 2 h, 4 h; VN: 6 h, 8 h; Col I: 2 h, 4 h; Col IV: 4 h, 8 h; Lam I: 2 h, 4 h). Cells that had migrated on the lower membrane surface were fixed in 4% formaldehyde and stained in 0.1% Crystal violet solution (200 mM Borat, pH 9) for 10 min. Cells left on the upper side of the Transwell membrane were removed and excess color washed away with water. Images were taken at 20 X magnification. Color was dissolved in 10% acetic acid for 5 min and read at 600 nm.

### Transwell invasion assay

CytoSelect™ 96-Well Cell Invasion Assays (Cellbiolabs, Cat#CBA-112, San Diego, CA, U.S.;) were performed according to the manufacturer’s protocol as previously described [45].

### Immunocytochemistry

Twenty thousand cells were allowed to grow for 48 h in complete medium in an 8-well chambered slide (Labtek II, 154534). Cells were washed in serum-free medium, fixed with 4% formaldehyde (Solvecco) and washed in TBST (25 mM Tris pH 7.4, 150 mM NaCl, 0.1% Tween 20) followed by permeabilization in 0.1% Triton X-100 (Sigma-Aldrich) for each 10 mins at R.T.. Unspecific binding was blocked in 1% BSA/TBST (Sigma-Aldrich) at R.T. for 1 h followed by incubation with primary and secondary antibodies for each 1 h at R.T.. Cells were after antibody incubations washed twice with TBST and finally stained with Hoechst (1:7500 dilutions, Life technologies) for 3 mins. The cells were washed, mounted in fluorescent mounting media (DAKO) and imaged using a Nikon A1+ confocal laser microscope system equipped with Nis Elements Advance research imaging software 4.3.0 (Nikon, Sweden, Stockholm) at 60 X. For quantification fluorescent signal intensity in 100–400 cells per experiment was measured with respect to a threshold set on experimental controls.

### Peptide level high-resolution isoelectric focusing (HiRIEF)

SILAC labeled peptide samples were prepared for quantitative phosphoproteomics and proteomics analysis of control cells and TRAP3^high^ cells, while TMT labeled peptide samples were prepared for scrambled and knockdown cells (sh2 and sh3 + 4) according to a detailed description in the Additional file 1: Material and Methods.

HiRIEF was performed as described previously for analysis of both SILAC and TMT labeled samples [47]. Immobilized pH gradient (IPG) gel strips (GE Healthcare Bio-Sciences AB, Uppsala, Sweden) with linear pH ranges of 2.5–3.7 (“ultra-acidic” range) and of 3–10 (“wide range”) were employed for standard phosphoproteomics and for proteomics analyses, respectively. Strips were divided into 72 fractions (fraction numbering proceeds from the acidic end towards the basic end of the strips), and extracted to V-bottom 96-well plates with a liquid handling robot (GE Healthcare prototype modified from Gilson liquid handler 215). Plates were lyophilized in a Speedvac prior to liquid chromatography-mass spectrometry (LC-MS) analysis.

### LC-MS data statistical analyses

The LC-MS proteomics data have been deposited to the ProteomeXchange Consortium via the PRIDE partner repository with the dataset identifier PXD006430. (https://www.ebi.ac.uk/pride/archive/login). Details regarding the LC-MS analysis and the calculations of protein and phospho-site ratios are described in Additional file 1: Material and Methods.

To define significantly regulated events in both SILAC-based or TMT-based quantitative analysis, a median absolute deviation (MAD) value for all the ratios in each experimental condition was calculated as the median absolute value of the differences between each individual log_2_(ratio) and the median log_2_(ratio). All log_2_ transformed ratios were median absolute deviation (MAD) scaled by calculating robust z-scores (MADs away from the median), as described before [48, 49], to correct for their skewed distributions. Significantly regulated events were those with robust z-scores of at least −/+ 2.5 and −/+ 3 in both replicates for SILAC-based phosphoproteomics and standard proteomics analysis respectively. For TMT-based standard proteomics analysis, significantly regulated events were those with average robust z-scores of at least −/+ 3 and t-test *p*-value <0.01 for both sh2 and sh3 + 4 samples.

### Bioinformatics analyses

Plots were generated using RStudio. Gene assignment to different classes was based on information retrieved from publicly available databases for protein kinases [50], protein phosphatases [51], transcription factors [52–54] and enzymes of the ubiquitin and ubiquitin-like (UBL) conjugation systems [55]. The list of human phosphorylation sites with a previously reported function was obtained from the PhosphositePlus database, released 2017–02-16 [56]. Gene ontology (GO) enrichment analysis was performed with the web service GOrilla by selecting the “Two unranked lists of genes” option and setting a *p*-value threshold of 10^−3^. The target set included all the genes that were significantly regulated in either of the proteomics and phosphoproteomics analyses (842 genes), while the background set was the list of all the identified genes (9570 genes).

Network analysis of the phosphorylated proteins was performed using the Cytoscape software platform. Significantly regulated genes from the GO analysis belonging to biological adhesion or ECM organization processes were employed to generate the network. Direct interactions between those genes were extracted from the STRING database (version 10.0, 2016–04-16) [57] and visualized with Cytoscape.

### Statistical analyses

Results visualized in columnar graphs were expressed as mean values ± standard deviation. Boxplots display the 25th and 75th percentile with medians as vertical line. Whiskers represent the minimum and maximum. Analysis and statistical comparison of the mean of at least three biological replicates was compared with GraphPad Prism 6 Software and each experiment based on one to five technical replicates. The respective amount of biological experiments was denoted more specifically in each figure legend. If not denoted specifically, TRAP-overexpressing cells were compared to control cells and TRAP knockdown cells compared to scrambled cells, respectively. In case of normal distribution, statistics was done by parametric two sample t-test (2 groups) or ANOVA test (> 2 groups). Otherwise, non-parametric Mann- Whitney test (2 groups) or Kruskal- Wallis one- way ANOVA on ranks (> 2 groups) was applied. A *p*-value <0.05 was considered significant (*); values *p* < 0.01 (**), values *p* < 0.001 (***), values *p* < 0.0001 (****) were marked in the graphs, respectively.

See Additional file 1: Material and Methods for description of gene expression analysis, cell lysis, SILAC labeling, protein extraction for mass spectrometric analyses, protein digestion, Tandem Mass Tag labeling, liquid chromatography tandem mass spectrometric analyses and proteomics database search, protein and phospho-peptide ratios calculation.

## Results

### Generation and characterization of TRAP-overexpressing and TRAP knockdown MDA-MB-231 cells

**Fig. 1:**
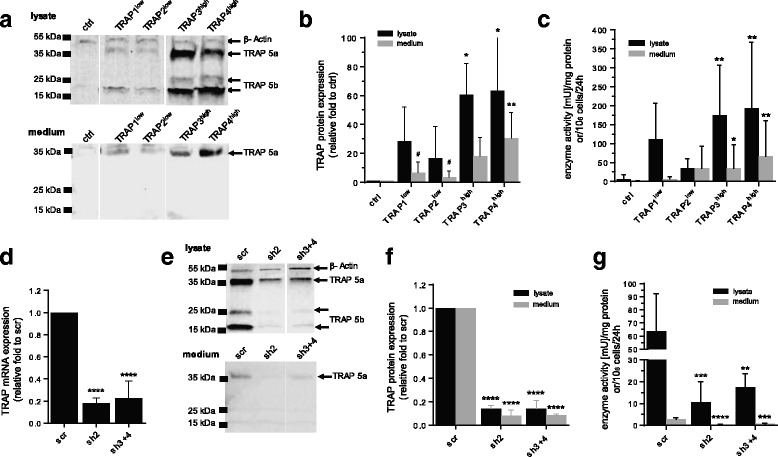
Generation and characterization of TRAP-overexpressing and TRAP knockdown MDA-MB-231 breast cancer cells. TRAP protein expression and enzymatic activity in MDA-MB-231 breast cancer cells overexpressing TRAP (TRAP1^low^, TRAP2^low^, TRAP3^high^, TRAP4^high^) compared to the mock-transfected control (ctrl) (**a**-**c**) and in TRAP knockdown cells (sh2 and sh3 + 4) compared to the scrambled control (scr) (**d**-**g**). TRAP knockdown was established by sh-hairpin technology in the TRAP3^high^ clone and initially tested by TRAP mRNA quantification (**d**, *n* = 3). One representative Western blot of TRAP staining of lysate and medium (monomeric TRAP 5a: 37 kDa, cleaved TRAP 5b: 16 kDa and 25 kDa) together with the normalization control β-Actin (42 kDa, only in lysate) is shown (**a**, **e**). Measurements in media are normalized to cell number and time in culture. Differential TRAP protein expression of the replicate means of biological replicates was quantified by densitometry (**b**, *n* = 3; **f**, *n* = 3). TRAP enzymatic activity, is normalized to total protein expression for lysates or number of cells and time in culture for medium (**c**, *n* = 7; **g**, *n* = 6). Statistical comparison was performed on biological replicates by Kruskal-Wallis test (Fig. 1**b**) or ANOVA test (Fig. 1**c**, **d**, **f**, **g**). Groups are generally compared to their respective controls (ctrl or scr) and significance is annotated with an asterisk (*). Specifically, the comparison of TRAP protein in medium between the high and low expressing cells TRAP^low^ and TRAP4^high^ is annotated with a hash (#). “n=” indicates the number of replicates

MDA-MB-231 cells, an invasive breast cancer cell line that has previously not been tested with regard to the role of TRAP was employed to test whether TRAP overexpression could further enhance invasive capabilities of these cells. As this cell line expresses low levels of TRAP, processing within the different cellular compartments, secretion, as well as signaling of TRAP in the overexpression system should be similar to the parental one. Four clonal populations of cells stably transfected with a full-length rat TRAP and a control cell population transfected with a mock insert (control) [46], were characterized by comparing TRAP protein expression level and enzymatic activity in cell lysates and media (Fig. 1a-c). As previously reported, in TRAP-overexpressing cells [46] both isoforms TRAP 5a and TRAP 5b are present in cell lysates, whereas only the monomeric isoform 5a is present in the media (Fig. 1a). TRAP-overexpressing cell populations possess intermediate (TRAP1^low^ and TRAP2^low^) and high (TRAP3^high^ and TRAP4^high^) TRAP protein levels and enzymatic activity in lysate and medium as compared to control cells (Fig. 1b, c).

Additionally, TRAP3^high^ cells were transfected with two different shRNA sequences targeting rat TRAP (sh2 and sh3 + 4) to generate TRAP knockdown cells. TRAP3^high^ cells transfected with a scrambled shRNA sequence (scr) were used as control (Fig. 1d-g). TRAP mRNA (Fig. 1d) and protein expression (Fig. 1e, f) were decreased by around 80% in TRAP knockdown cell lysates and media. TRAP enzymatic activity was decreased in both scrambled cells and TRAP knockdown cells compared to TRAP3^high^ cells, however significantly lower in TRAP knockdown cells compared to scrambled cells (Fig. 1g).

### Overexpression of TRAP increases cell growth and proliferation

**Fig. 2:**
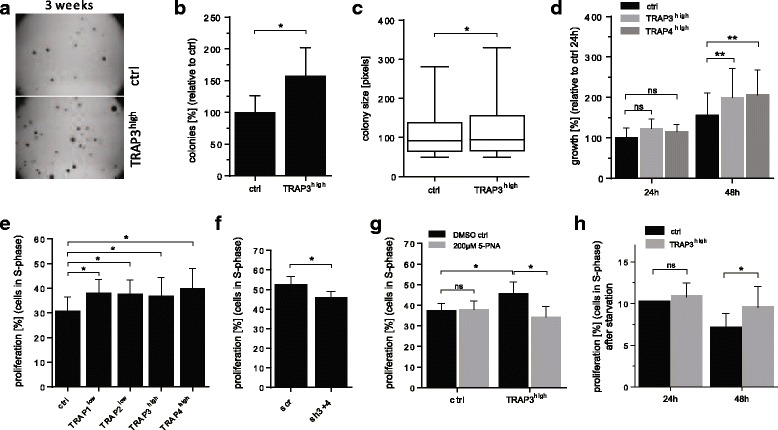
TRAP increases growth and proliferation. Assessment of the impact of TRAP on anchorage-independent growth of TRAP-overexpressing TRAP3^high^ cells (**a**-**c**, *n* = 4). One representative well with colonies is shown in (**a**). After 3 weeks of culture colony number (**b**) and colony size (**c**) were measured. Anchorage-dependent growth of TRAP-overexpressing clone TRAP3^high^ is assessed by spectral quantification of adhered cells cultured for 24 h and 48 h in complete medium (**d**, *n* = 3). Measurement of proliferation is quantified based on the incorporation of EdU in the DNA during S-phase, immunocytochemistry and subsequent image analysis (**e**-**h**). Assessment of proliferation in TRAP-overexpressing (**e**, *n* = 6–14) and in the TRAP knockdown cells (**f**, *n* = 3–5) after 24 h culture in complete medium. Proliferation is also quantified in control cells (ctrl) and in TRAP-overexpressing TRAP3^high^ cells after treatment with the small molecule TRAP inhibitor 5-PNA (200 μM) for 24 h (**g**, *n* = 4) and after starvation for 24 h and 48 h (**h**, *n* = 3). Statistical comparison was performed on biological replicates by t-test (Fig. 2**b**, **f**), Mann-Whitney test (Fig. 2 **c**) or ANOVA test (Fig. 2**d**, **e**, **g**, **h**). Groups are generally compared to their respective controls (ctrl or scr, DMSO treated) or indicated with brackets and significance is annotated with an asterisk (*). ns = non-significant. “n=” indicates the number of replicates

Anchorage-independent growth of TRAP3^high^ cells was assayed by their ability to form colonies in soft agar (Fig. 2a, one representative well per condition). Numbers of colonies were increased by 58% in TRAP3^high^ cells compared to control cells (Fig. 2b) and significantly larger than the ones formed by control cells (Fig. 2c). Anchorage-dependent growth of TRAP3^high^ and TRAP4^high^ cells compared to control cells were increased by 44% and 50%, respectively, after 48 h of culture in serum-supplemented medium (Fig. 2d). Proliferation, determined by the incorporation of the thymidine analogue EdU during S-phase, was increased from 31% in the control cells to 37%–43% in the TRAP-overexpressing cells (Fig. 2e). Accordingly, TRAP knockdown cells (sh3 + 4) displayed significantly lower proliferation than scrambled cells (Fig. 2f). Additionally, inhibition of TRAP by the small molecule inhibitor 5-PNA normalized proliferation of the TRAP3^high^ cells down to the level of the control cells, without affecting proliferation of the control cells (Fig. 2g). Interestingly, TRAP3^high^ cells also displayed an increased capacity to proliferate in serum-free medium after 48 h (Fig. 2h), where around 9.5% of the TRAP3^high^ cells remained cycling compared to 7% of the control cells.

### TRAP enhances an elongated morphology and metastasis-related hallmarks

**Fig. 3:**
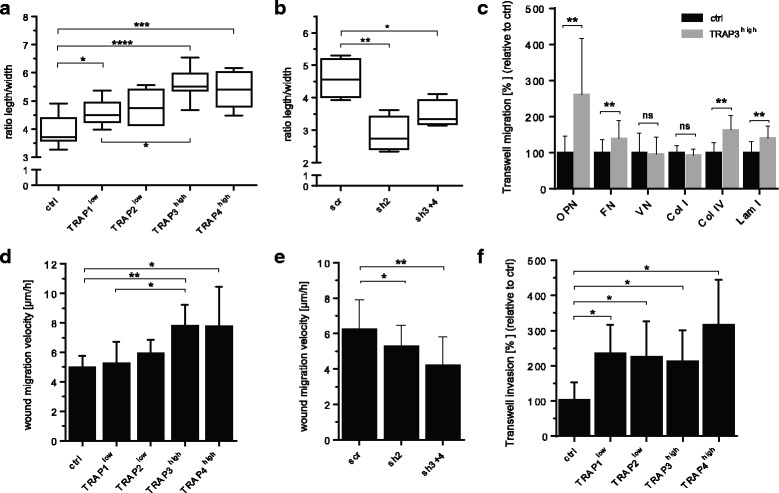
TRAP promotes an elongated morphology, migration and invasion. Impact of TRAP expression on functional features associated with cancer progression. Morphological analysis was performed on TRAP-overexpressing cells (**a**, *n* = 9) and TRAP knockdown cells (**b**, *n* = 4) and respective controls (ctrl or scr; >200 cells per condition) by measurement of length-to-width ratios in ImageJ program. Transwell migration derived from differences at two time points was assessed on inserts precoated with 10 μg/mL of the matrix and basement membrane proteins osteopontin (OPN, *n* = 9), fibronectin (FN, *n* = 9), collagen type I (Col I, *n* = 10), collagen type IV (Col IV, *n* = 10), vitronectin (VN, *n* = 5), and laminin type I (Lam I, *n* = 5) in serum-free medium (**c**). Wound migration experiments were performed by live cell imaging over 30 h of TRAP-overexpressing cells (**d**, *n* = 3–6) and TRAP knockdown cells (**e**, *n* = 5) in serum-free medium. Transwell invasion through precoated basement membrane matrix, and induced by a serum gradient, was allowed to proceed for 18 h for all TRAP-overexpressing cell lines (**f**, *n* = 4). Statistical comparison was performed on biological replicates by ANOVA test (Fig. 3**a**, **b**, **d**–**f**) or t-test (Fig. 3 **c**). Groups are generally compared to their respective controls (ctrl or scr) or indicated with brackets and significance is annotated with an asterisk (*). ns = non-significant. “n=” indicates the number of replicates

TRAP affects cellular morphology as cells with increasing TRAP protein levels showed increasingly elongated phenotypes. Cellular length-to-width ratios were increased in the TRAP-overexpressing (TRAP1^low^, TRAP3^high^, TRAP4^high^) cells compared to control cells. Additionally, TRAP3^high^ cells were more elongated than the TRAP1^low^ cells (Fig. 3a). This morphological phenotype was reverted upon TRAP downregulation, as shown by decreased length-to-width ratios in sh2 and sh3 + 4 cells compared to scrambled cells (Fig. 3b). Furthermore, transwell migration was higher in TRAP3^high^ cells than in control cells when the wells were coated with fibronectin (1.4-fold), collagen IV (1.6-fold) and laminin I (1.4-fold) (Fig. 3c). Interestingly, a very prominent increase in transwell migration was observed in the presence of the phosphorylated extracellular matrix (ECM) protein osteopontin (OPN) (2.6-fold), a well-known substrate for TRAP [10]. No difference in transwell migration was detected in the presence of vitronectin or collagen I. Furthermore, wound migration was assessed by live cell imaging over 30 h. TRAP^high^ cells but not TRAP^low^ cells displayed significantly increased migration velocity compared to the control cells; additionally migration velocity was significantly increased in TRAP3^high^ cells compared to TRAP1^low^ cells, indicating a level-dependent regulation of migration by TRAP (Fig. 3d). Accordingly, migration velocity was decreased in sh2 and sh3 + 4 cells compared to scrambled cells (Fig. 3e). Finally, TRAP also promoted transwell invasion through a basement membrane layer, as indicated by a 2.3–3.3-fold increase in invasion in TRAP-overexpressing cells as compared to control cells (Fig. 3f). Importantly, treatment with the TRAP inhibitor 5-PNA was recently shown to revert TRAP-dependent promotion of cell wound migration and transwell invasion [45].

### Proteomics and phosphoproteomics profiling of TRAP perturbed cells

In order to investigate the molecular changes underlying the phenotype of TRAP-overexpressing cells, a large-scale quantitative proteomics and phosphoproteomics analyses using high-resolution isoelectric focusing (HiRIEF) fractionation [47] coupled to LC-MS was performed. Analyses were conducted in biological duplicates and relative quantifications are expressed as ratios of TRAP3^high^ (Heavy SILAC labeled) relative to control (Light SILAC labeled) samples.

**Fig. 4:**
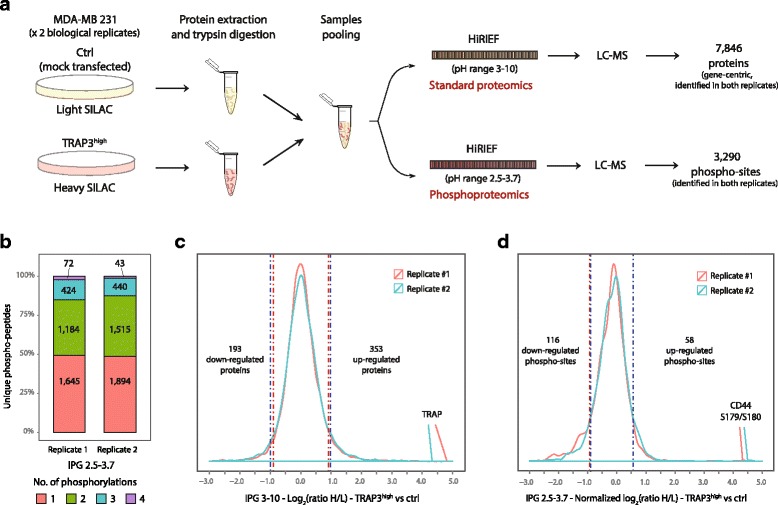
Proteomics and phosphoproteomics profiling of TRAP-overexpressing cells. Experimental workflow employed to analyze the proteome and phosphoproteome of control (ctrl, Light SILAC labeled) and TRAP3^high^ (Heavy SILAC labeled) MDA-MB-231 cells (*n* = 2). Protein extracts were digested with trypsin and then equal amounts of Light and Heavy peptides were pooled. Peptides were fractionated by HiRIEF using immobilized pH gradient (IPG) strips with pH range 3–10 for proteomics analysis, and with pH range 2.5–3.7 for phosphoproteomics analysis, prior to LC-MS analysis (**a**). Number of unique phospho-peptides identified in each biological replicate, broken down by number of phosphorylations (**b**). Protein log_2_(ratio H/L) (**c**) and phosphorylation sites normalized log_2_(ratio H/L) (**d**) distribution for the two biological replicates. A threshold for significance was set at −/+3 MAD and −/+ 2.5 MAD away from the median for standard proteomics and phosphoproteomics analysis respectively. “n=” indicates the number of replicates

The proteomic analysis resulted in the identification and quantification of 7957 proteins corresponding to 7846 genes in both replicates (Fig. 4a, Table 1).

**Table 1:** Analysis conditions and number of identifications for each experimental approach

	TRAP3^high^ vs control				TRAP shRNA
	Phosphoproteomics analysis Ultra-acidic (IPG 2.5–3.7)		Standard proteomics analysis Wide-range (IPG 3–10)		Wide-range (IPG 3–10)
	Replicate #1	Replicate #2	Replicate #1	Replicate #2	
Nr. of unique peptides	6266	5771	79,410	81,851	97,153
Nr. of unique phospho-peptides	3325	3892	b	b	b
Nr. of unique proteins	1,704^a^	1,968^a^	8433	8447	9848
Nr. of unique genes	1,695^a^	1,957^a^	8309	8315	9189

^a^For phosphoproteomics analysis the reported numbers refer only to proteins found to carry phosphorylated amino acids, and their corresponding genes

^b^No phospho-peptides are reported because phosphorylation was not included as dynamic modification when searching the data

Since TRAP possesses phosphatase activity, phosphoproteomics analysis upon TRAP overexpression was employed to identify putative TRAP targets. For phosphoproteomics analysis, HiRIEF fractionation using “ultra-acidic” pH range (2.5–3.7) IPG strips was employed to enrich for phosphorylated peptides, as the addition of phosphate groups decreases the peptide pI. This approach led to the identification of 6266 and 5771 unique peptides in replicate 1 and replicate 2, respectively, of which 3325 and 3892 were phosphorylated (Table 1). Across both replicates, 3290 unique phosphorylations sites corresponding to 1059 genes were identified (Fig. 4a).

Notably, these results demonstrate that HiRIEF fractionation alone can be used to perform phosphoproteomics profiling with moderate analytical depth, independently than other enrichment methods, as previously reported [58]. This approach is particularly suitable to identify multiply phosphorylated peptides, as about 50% of the identified phospho-peptides carry two or more phosphorylations. Multiply phosphorylated peptides are enriched in the first 30 fractions (acidic end) of the IPG 2.5–3.7 strip, while singly phosphorylated peptides are identified mostly in the more basic strip fractions (Fig. 4b; Additional file 2: Figure S1).

Phospho-site ratios (normalized to the total protein levels) and protein ratios have Pearson coefficients of correlation between replicates of 0.60 and 0.84 respectively (Additional file 3: Figure S2). One hundred ninety-three and 353 proteins and 116 and 58 phosphorylation sites were significantly down- and upregulated in both TRAP3^high^ replicates (Fig. 4c, d; Additional file 4: Table S1). Among the top upregulated phosphorylation sites, S179, S180 and S183 of CD44 displayed a much higher magnitude of regulation than any other sites, with an increase of more than 11-fold in TRAP3^high^ cells compared to control cells. These three sites located in the intracellular portion of CD44 do not have any previously reported function. The 116 phosphorylation sites downregulated in response to TRAP overexpression represent putative targets of TRAP phosphatase activity. Among the significantly downregulated phosphorylation sites, 11 sites were previously annotated to be functional, including S183 of protein phosphatase 1G (PPM1G); S131 and S137 of lysine-specific histone demethylase 1A (KDM1A); S957 and S966 of structural maintenance of chromosomes protein 1A (SMC1A); S27 of isoform 2 of X-ray repair cross-complementing protein 6 (XRCC6); and S453 and T455 of isoform 3 of Mediator of DNA damage checkpoint protein 1 (MDC1). All these sites are involved in promotion of DNA damage response. Finally, the list of genes corresponding to the significantly regulated phosphorylation sites is enriched in GO Molecular Function terms “cell adhesion molecule binding” and “cadherin binding”, as well as Cellular Component terms related to cellular junctions (Additional file 5: Figure S3).

**Fig. 5:**
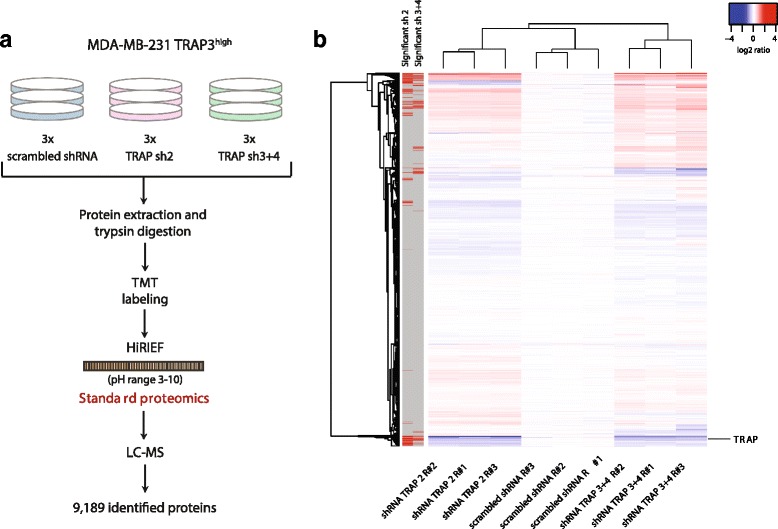
Proteomics and phosphoproteomics profiling of TRAP knockdown cells. Experimental workflow applied to perform standard proteomics analysis of TRAP3^high^ MDA-MB-231 cells transfected with a shRNA with scrambled sequence (scr, *n* = 4) or with two different shRNAs targeting TRAP (sh2 and sh3 + 4, *n* = 3 each) (**a**). Protein extracts from each condition were digested to peptides with trypsin, labeled with Tandem Mass Tags (TMT), pooled, fractionated by HiRIEF using a wide-range (pH 3–10) IPG strip and analyzed by LC-MS. Heatmap representing complete linkage hierarchical clustering based on Euclidian distance of protein ratios measured in the proteomics analysis (**b**). Ratios are represented for each sample relative to the average of the scrambled shRNA samples (**b**). Row color-coding indicates significantly regulated proteins (red colored), defined by a log_2_(ratio) of more than −3/+3 MAD away from the median and t-test *p*-value <0.01. “n=” indicates the number of replicates

Additionally quantitative proteomics analysis by tandem mass tags (TMT) of scrambled and TRAP knockdown (sh2 and sh3 + 4) cells was performed in three biological replicates each (Fig. 5a), identifying and quantifying 9848 proteins corresponding to 9189 genes. Quantifications for each TMT channel are expressed as ratios relative to the average of the three scrambled cell samples. Hierarchical clustering based on Euclidian distance and complete linkage separates samples based on experimental condition, illustrating good reproducibility of replicate measurements (Fig. 5b). 174 and 144 proteins were significantly down- and upregulated respectively, upon TRAP knockdown in both sh2 and sh3 + 4 samples (Additional file 6: Figure S4; marked with red row-side colors in Fig. 5b). TRAP protein expression level was significantly reduced by at least 1.5-fold in sh2 and sh3 + 4 cells.

### Proteomics and phosphoproteomics analyses of TRAP-perturbed cells reveal regulation of cell adhesion and extracellular matrix organization network

Enrichment of gene ontology (GO) processes was evaluated comparing the list of all genes regulated upon TRAP perturbation in either of the phosphoproteomics or proteomics analyses (Additional file 7: Table S2) with the list of all identified genes. Significantly regulated genes included 21 protein kinases, 9 protein phosphatases, 25 transcription factors and 24 ubiquitin and ubiquitin-like (UBL)-conjugating system enzymes (Additional file 7: Table S2).

**Fig. 6:**
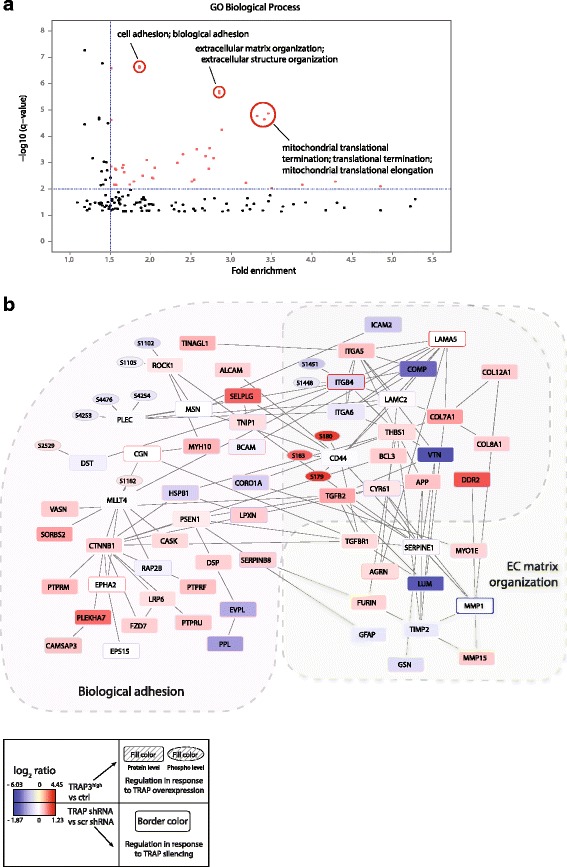
Phosphoproteomic and proteomic analyses of TRAP-perturbed cells reveal regulation of cell adhesion and extracellular matrix organization network. GO Biological Process enrichment analysis of all the genes regulated upon TRAP perturbation (842 genes, Additional file 7: Table S2) compared to all the identified genes (9570 genes) (**a**). Enriched GO terms are displayed by significance of the enrichment (q-value, multiple testing corrected) and fold enrichment. Interaction network of proteins belonging to the GO terms “biological adhesion” and “extracellular matrix organization” and significantly regulated upon TRAP perturbation (**b**); interactions were retrieved from the STRING database. Squared shaped nodes represent proteins and round shaped nodes represent phosphorylation sites. Nodes fill color: log_2_ transformed ratio (H/L) values for standard proteomics and phosphoproteomics analysis of TRAP3^high^ cells (Heavy SILAC labeled) compared to control cells (Light SILAC labeled) (replicate 1 and 2 average). Nodes border color: log_2_ transformed ratio values for standard proteomics analysis of scrambled, TRAP sh2 and sh3 + 4 cells (average of sh2 and sh3 + 4 ratios relative to scrambled)

Biological adhesion and ECM organization processes showed a high degree of enrichment based on significance and fold enrichment (Fig. 6a). This result is in agreement with our previous observations of increased migration and invasion upon TRAP overexpression, as those processes are tightly interconnected. Additionally, we observed enrichment in processes related to mitochondrial translational termination; examination of the genes included in these processes shows that several mitochondrial ribosomal proteins were upregulated upon TRAP knockdown. As mitochondrial ribosomes translate exclusively mitochondrial encoded mRNA (13 genes, mainly electron transport chain proteins), this result might indicate increased mitochondrial protein synthesis in the mitochondria upon TRAP knockdown, possibly suggesting that TRAP affects cellular metabolism.

To examine the regulation of the genes specifically involved in modulating biological adhesion and ECM organization upon TRAP perturbation, we generated a protein-protein interaction network of TRAP regulated proteins classified into those GO terms, using the STRING database [57] (Fig. 6b). Proteins in the network involved with cell adhesion include protein kinases ephrin type-A receptor 2 (EPHA2), peripheral plasma membrane protein (CASK) and ROCK1 as well as receptor-type tyrosine-protein phosphatases mu, F and U (PTPRM, PTPRF and PTPRU). Interestingly, several proteins known to be involved in the migration and invasion processes were included in the network, such as matrix metalloproteases (MMPs), collagens (core components of the ECM), integrins, CD44, TGFβ2 and TβR1.

Based on previous publications reporting a connection of TRAP with TGFβ signaling through TRIP-1 and the significant upregulation of CD44 phosphorylation sites and the TGFβ pathway-associated proteins TGFβ2, TβR1 and SMAD2 measured in the SILAC proteomics analysis (Table 2), we hypothesized that an activation of TGFβ signaling or regulation of CD44 upon TRAP overexpression is responsible for the observed metastasis-related properties.

**Table 2:** Expression level of TGFβ pathway related proteins in TRAP-overexpressing MDA-MB-231 cells

		TRAP3^high^ vs control		
Gene name	Description	Protein log2(ratio H/L) – Replicates average	Fold change (H/L) – Replicates average	Significance^a^
TβR1	TGF-beta receptor type-1	1.15	2.22	Significantly reg.
TβR2	TGF-beta receptor type-2	0.97	1.95	ns
TβR3	TGF-beta receptor type-3	0.69	1.62	ns
TGFβ1	Transforming growth factor beta-1	−0.27	0.83	ns
TGFβ2	Transforming growth factor beta-2	2.25	4.77	Significantly reg.
TGFβ1I1	Isoform 2 of Transforming growth factor beta-1-induced transcript 1 protein	−0.42	0.75	ns
TGFβ I	Transforming growth factor-beta-induced protein ig-h3	0.45	1.36	ns
TGIF1	TGFB-induced factor homeobox 1	0.80	1.75	ns
TGIF2	TGFB-induced factor homeobox 2	−0.76	0.59	ns
SMAD1	Mothers against decapentaplegic homolog 1	0.19	1.14	ns
SMAD2	Mothers against decapentaplegic homolog 2	1.34	2.52	Significantly reg.
SMAD3	Mothers against decapentaplegic homolog 3	0.19	1.14	ns
SMAD4	Mothers against decapentaplegic homolog 4	0.78	1.72	ns
SMAD5	Mothers against decapentaplegic homolog 5	−0.19	0.87	ns

^a^Significance describes whether the level of the indicated protein is significantly altered in TRAP3^high^ compared to control cells, based on cutoffs for log_2_(ratio) of −/+ 2.5 MAD away from the median (see Material and methods). “ns” indicates that the measured protein level is not significantly changed in TRAP3^high^ cells

### TRAP effects on proliferation and migration are mediated via TGFβ pathway-associated proteins

**Fig. 7:**
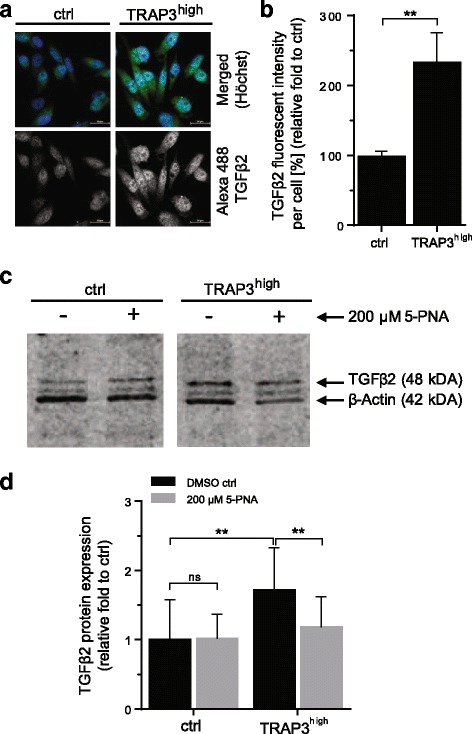
TRAP overexpression upregulates TGFβ-pathway associated proteins. Expression of TGFβ2, respectively after 24 h treatment with the small molecule TRAP inhibitor 5-PNA (200 μM) or respective control in complete medium. Quantification of Immunocytochemistry (**a**, **b**, *n* = 3) and Western blotting (**c**, **d**, *n* = 4). One representative image of immunocytochemistry staining (**a**, TGFβ2, green; Hoechst, blue) or blot (**c**, TGFβ2, 48 kDa; normalization control β-Actin 42 kDa) is shown. Quantification was performed by analysis of fluorescent intensities per cell for immunocytochemistry (**b**) and by densitometry for western blot analysis (**d**). Statistical comparison was performed on biological replicates by or t-test (Fig. 7 **b**) or ANOVA test (Fig. 7 **d**). Groups are generally compared to their respective controls (ctrl, untreated) or indicated with brackets and significance is annotated with an asterisk (*). ns = non-significant. “n=” indicates the number of replicates

To consolidate our hypothesis, protein expression of the most upregulated TGFβ pathway protein, TGFβ2, was quantified in TRAP3^high^ cells and compared to control cells (Fig. 7). Concordantly with the global analysis, TGFβ2 protein was upregulated in TRAP3^high^ cells as shown by both immunocytochemistry (Fig. 7a, b) and Western blotting (Fig. 7c, d). Additionally, TGFβ2 expression was reduced to control cell level upon inhibition with the TRAP inhibitor 5-PNA (Fig. 7c, d).

**Fig. 8:**
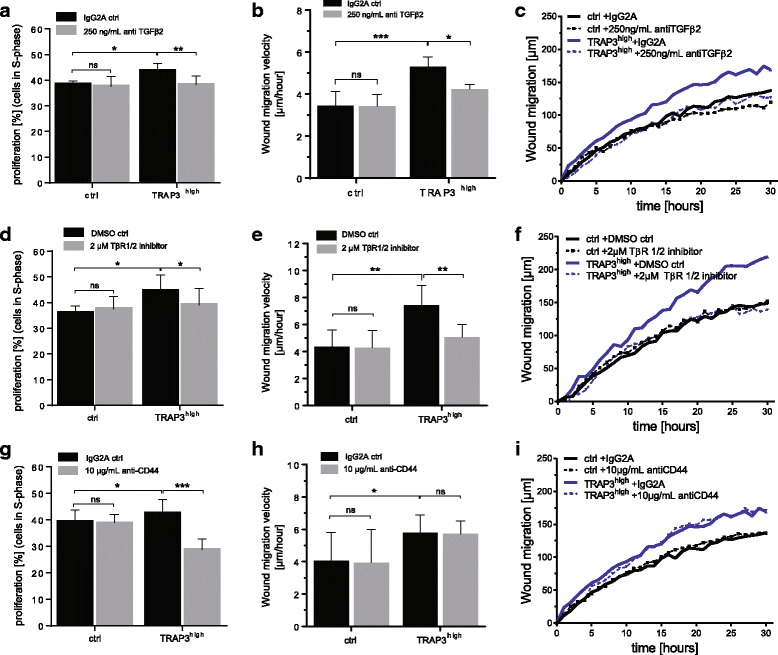
TRAP-dependent proliferation and migration are mediated via the TGFβ2/TβR and CD44. Functional analysis after treatment with a blocking antibody anti-TGFβ2 (0.25 μg/mL, **a**-**c**, *n* = 6), the small molecule inhibitor LY2109761 against TGFβ receptor type 1/type 2 kinase activity (2 μM, **d**-**f**, *n* = 4) or with a blocking antibody anti-CD44 (10 μg/mL, **g**-**i**, *n* = 3) and respective controls. Proliferation was assessed after 24 h treatment in complete medium (**a**, **d**, **g**). Live cell imaging was performed over 30 h in serum-free medium containing blocking antibody or small chemical inhibitor and wound migration velocity (**b**, **e**, **h**) and migration curves (**c**, **f**, **i**) are compared. Statistical comparison was performed on biological replicates by ANOVA test. Groups are generally compared to their respective controls (ctrl, untreated) or indicated with brackets and significance is annotated with an asterisk (*). ns = non-significant. “n=” indicates the number of replicates

To examine the functional impact of TGFβ2/TβR in TRAP3^high^ cells, we assayed cell proliferation and migration upon treatment with blocking antibodies against TGFβ2 (Fig. 8a-c) or with a small molecule inhibiting TβR1/2 (LY2109761; Fig. 8d-f). Blocking of either TGFβ2 or TβR1/2 kinase activity reduced the proliferation of TRAP3^high^ cells to the level of control cells (Fig. 8a, d). Furthermore, interfering with the TGFβ pathway-associated proteins decreased migration (Fig. 8b, c, e, f) in TRAP3^high^ cells to the levels detected in the control cells. Migration curves over 30 h are shown in Fig. 8c and f. Control cell proliferation or migration were not affected when interfering with the TGFβ pathway associated proteins by using blocking antibodies against TGFβ2 or the small molecule inhibiting TβR1/2 activity (Fig. 8a-f). Treatment with the closely related ligand TGFβ1 did not affect proliferation and migration of control and TRAP3^high^ cells (Additional file 8: Figure S5).

As discovered by phosphoproteomics analysis, phosphorylation of S179, S180 and S183 of CD44 was increased more than 11-fold in TRAP3^high^ compared to control cells, representing the top upregulated phospho-events in TRAP3^high^ cells. These three phosphorylation sites are located in the intracellular domain of CD44 and do not have any previously reported function. No antibodies targeting those phosphorylation sites are currently available, thus we probed the role of CD44 in determining the TRAP-dependent phenotype by inhibiting CD44 using a blocking antibody (Fig. 8g-i). Upon anti-CD44 treatment, the proliferation rate of TRAP3^high^ cells was reduced to a level lower than that of control cells (Fig. 8g), while the TRAP-dependent increase in migration was not affected (Fig. 8h, i).

## Discussion

TRAP has been shown to be associated with tumor progression in several types of cancer and suggested to be clinically relevant as a marker for peritoneal dissemination in gastric cancer [20–22]. Recently, TRAP was identified as a pro-invasion oncogene and a prognostic marker in melanoma [20]. Moreover, TRAP’s functional role in invasion, cell motility and metastasis was suggested to be mediated through phosphorylation of focal adhesion complexes [20].

In this study, we investigated the effects of TRAP expression on cell properties related to the development of metastasis and on the cellular signaling of the MDA-MB-231 breast cancer cell line at the system level. The small molecule 5-phenylnicotinic acid (5-PNA), recently identified as a specific inhibitor of TRAP activity and TRAP-dependent migration and invasion, was employed as a tool to investigate the molecular events mediated by TRAP [45, 59]. We demonstrate that TRAP regulates metastasis-related features such as anchorage-independent and –dependent cell growth, proliferation, migration and invasion. Global proteomics and phosphoproteomic analyses showed that regulated events upon TRAP perturbation are mainly proteins and phosphorylation sites involved with cellular adhesion and extracellular matrix (ECM) organization. Based on these analyses and on the literature, we hypothesized that TGFβ pathway-associated proteins and three previously unreported intracellular phosphorylation sites of CD44 mediate the observed TRAP-dependent cellular phenotypic properties.

TRAP has been proposed as a differentiation and growth factor for cells of hematopoietic origin [60]. Effects of TRAP expression on cell transformation and tumor progression have been clinically validated in melanoma, as well as proven in vitro by an anchorage-independent growth assay [20]. TRAP overexpression correlates with increased tumor size and poor differentiation in hepatocellular cancer [21]. We consolidated and expanded these findings by using the breast cancer cell line MDA-MB-231, showing that TRAP overexpression increases cell growth as well as colony formation and cell proliferation above basal levels. Moreover, a higher number of TRAP-overexpressing cells compared to control cells were actively proliferating after 48 h serum starvation, indicating a lower requirement for exogenous growth stimulation.

Morphological changes such as cell rounding or cell spreading upon perturbation of TRAP expression have been reported in melanoma cells [20]. Modulation of TRAP expression was shown to impact the migration and invasion of melanoma and hepatocellular carcinoma cells both in vitro and in vivo*,* when either non-invasive cancer cells, expressing low amounts of TRAP or metastatic high-TRAP expressing cells were subjected to upregulation or knockdown, respectively [20, 21]. Moreover, also in non-malignant epithelial cells TRAP expression was linked to a regulation of cell migration [61]. This study demonstrates that TRAP overexpression enhances the elongated phenotype, migration and invasion capabilities of invasive breast cancer cells. Importantly, the elongated morphology and migration were regulated by TRAP in a dose-dependent manner. The presence of ECM proteins and basement membrane proteins Collagen IV and Laminin I increased transwell migration of TRAP3^high^ cells as compared to control cells, underscoring the role of TRAP during the invasive process. Transwell migration was particularly increased in the presence of osteopontin (OPN), a highly phosphorylated ECM protein previously suggested to be a physiological substrate for TRAP [10], and involved in the progression of TRAP-related pathologies such as the immuno-osseous disorder Spondyloenchondrodysplasia [9, 62]. OPN has been reported as a ligand to the CD44 receptor [41] and was shown to increase osteoclast migration [8], which is blunted upon antibody-mediated blocking of CD44 [63]. Inhibition of TRAP by the small molecule inhibitor 5-PNA was previously reported to decrease TRAP3^high^ cells migration and invasion [45]; here we showed that also proliferation of TRAP3^high^ cells is reduced to basal levels upon treatment with 5-PNA, altogether providing evidence that the above mentioned phenotypes of TRAP-overexpressing MDA-MB-231 cells are attributable to the overexpression of TRAP.

In parallel, global proteomics analysis of TRAP3^high^ cells revealed regulation of various proteins belonging to the GO terms “biological adhesion” and “ECM organization”. Coherently, an increase in migration and invasion on various ECM and basement membrane proteins was observed in the TRAP3^high^ cells. Enrichment in closely related GO terms, such as “cell adhesion molecule binding” and “cell junction”, was noted when analyzing phosphosites regulated in TRAP-overexpressing cells compared to control cells, further substantiating the involvement of TRAP in these functions. The list of 119 phosphorylation sites downregulated upon TRAP overexpression represent an inventory of putative targets of TRAP phosphatase activity or possible signaling intermediates; among those, eight sites with known regulatory function are involved in DNA damage response, another hallmark of cancer.

Most importantly, we identified a regulation of the TGFβ pathway-associated proteins TGFβ2, TβR1 and SMAD2, as well as a highly significant upregulation of previously unreported phosphorylation sites of CD44 upon TRAP perturbation in the MDA-MB-231 breast cancer cell line. Quantification of expression levels by several methodological approaches confirmed the upregulation of the ligand TGFβ2, which could be reverted by treatment with the TRAP inhibitor 5-PNA. Functional blocking of TGFβ2 or inhibition of TβR1/2 kinase activity restrained the increase in migration and proliferation promoted by TRAP. Antibody-mediated inhibition of CD44 reduced proliferation beyond the basal level of control cells.

Several reports support the concept that TGFβ promotes or restrains cell proliferation depending on the context [28]. TGFβ inhibited the proliferation of most epithelial cells and its growth inhibitory effect could be partially reverted by treatment with a specific inhibitor [64–66]. Poorly differentiated prostate cancer cells were resistant to TGFβ growth inhibitory effect in vivo [67]*.* Upon functional blocking of TGFβ2 or inhibition of TβR1/2 kinase activity, we observed decreased proliferation of TRAP-overexpressing cells but not of control MDA-MB-231 cells. Such lack of response in the control cells suggests that the malignant MDA-MB-231 cells are resistant to the growth-inhibitory effect of TGFβ, and that TRAP-dependent increase in proliferation of TRAP-overexpressing cells is TGFβ2-mediated. Furthermore, the invasive capacity of malignant breast cancer cells is enhanced by TGFβ1 [68] and inactivation of TGFβ signaling inhibited invasiveness in vitro and in vivo for colon carcinoma cell lines [69]. Additionally, here we detected a TRAP-dependent promotion of migration mediated by TGFβ2.

We could exclude a possible contribution of TGFβ1 because treatment with this TGFβ ligand modulated neither migration nor proliferation, as reported previously, despite constitutive expression of its receptors [68]. Thereby we substantiated that the TGFβ2 isoform is crucial for the TRAP-mediated effects. In support to this notion, TGFβ2 was previously attributed a dominant role in predicting the outcome of breast cancers [38] and aberrant expression of the TGFβ2 isoform exclusively was induced through an autocrine loop in glioma [39].

We were also interested in the regulation of CD44, as it has previously been reported to be connected to TGFβ pathway [70–72] and to be phosphorylated by TRβ1 [40]. Our analysis identified three phosphorylation sites of CD44 as the top upregulated phosphorylation events in TRAP3^high^ cells. Additionally, CD44 is a receptor for OPN [41], which is the protein that prominently increased transwell migration in TRAP-overexpressing cells in this study. Functional blocking of CD44 resulted in a decrease of proliferation of TRAP3^high^ cells beyond the basal level of control cells, but had no effect on cell migration. This suggests that cell migration is regulated by TRAP via TGFβ independently of CD44, but that the basal and TRAP-dependent proliferative activity of the TRAP3^high^ cells is regulated through both TGFβ signaling and CD44.

As this study is performed in vitro and uses a metastatic cell line, similar experiments in other cancer cell lines at different stages of metastatic progression would be crucial to fully delineate the effects of TRAP, possibly revealing a role in promoting metastasis of non-invasive cell lines as well [20, 21]. Additionally, knockdown of TRAP in invasive cancer cells expressing high TRAP levels might be employed to test whether their invasive phenotype is TRAP-dependent. Limitations include also a full dissection of the respective TGFβ pathway and a reconfirmation, to allow for a generalization to other TRAP expressing cell lines.

## Conclusion

This is the first study investigating the effects of TRAP perturbation in cancer cells on a global scale and identifying possible substrates and signaling intermediates. Herein, we show that TRAP promotes metastasis-related cellular properties, such as cancer cell proliferation, migration and invasion beyond basal levels in malignant breast cancer cells and regulates cell adhesion and extracellular matrix (ECM) organization. TRAP-dependent migration and proliferation can be neutralized upon inhibition of TGFβ2/TRβ and CD44 or inhibition of TRAP by the small molecule 5-PNA. Altogether, this data provide the basis for further studies investigating TRAP signaling and a novel possible targeting strategy for the treatment of tumors with high TRAP expression.

## Additional files

Additional file 1: Material and Methods. Additional explanations on Material and Methods including descriptions of gene expression analysis, cell lysis, SILAC labeling, protein extraction for mass spectrometric analyses, protein digestion, Tandem Mass Tag labeling, liquid chromatography tandem mass spectrometric analyses and proteomics database search, protein and phospho-peptide ratios calculation. (DOCX 32 kb)

Additional file 2: Figure S1. Distribution of unique peptides across fractions generated by HiRIEF separation on IPG 2.5–3.7 strips. Number of unique peptides and phospho-peptides identified across fractions in each biological replicate (A) and (B), displayed by number of phosphorylations. Fraction numbering proceeds from the acidic end towards the basic end of the strips. Multiply phosphorylated peptides are identified prevalently in the first 30 fractions in the IPG 2.5–3.7 strip. (PDF 148 kb)

Additional file 3: Figure S2. Quantitative reproducibility of biological replicates employed for phosphoproteomics and proteomics analysis of TRAP3^high^ (Heavy SILAC labeled) and control (Light SILAC labeled) MDA-MB-231 cells. Correlation of log_2_ transformed ratio (H/L) values for replicate pairs for phosphoproteomics analysis (A) and standard proteomics analysis (B); Pearson correlation coefficient is displayed. (PDF 2872 kb)

Additional file 4: Table S1. Phosphorylation sites significantly regulated in TRAP3^high^ cells compared to control (ctrl) cells. Unique phosphorylation sites are defined by a sequence window of 15 amino acids centered at the phosphorylated residue. The column “Protein class” describes to which of the examined protein class a protein belongs to. Examined proteins classes include protein kinases, protein phosphatases, ubiquitin and UBL system enzymes and transcription factors. The column “Functional site” describes whether the phospho-site has a known regulatory function based on the information available on the database PhosphoSitePlus. (XLSX 48 kb)

Additional file 5: Figure S3. Volcano plots representing the selection of proteins significantly regulated upon TRAP knockdown in TRAP3^high^ MDA-MB-231 cells. The average log_2_ transformed ratio of the three replicates is plotted for each experimental condition. Significantly regulated events are defined by log_2_ transformed ratios at least −/+3 MAD away from the median and *p*-values lower than 0.01. TRAP sh2 samples versus scrambled (A). TRAP sh3 + 4 samples vs scrambled (B). (PDF 5171 kb)

Additional file 6: Figure S4. GO enrichment analyses of genes corresponding to phosphorylation sites significantly regulated upon TRAP overexpression in MDA-MB-231 cells. GO terms enrichment was evaluated for the set of genes corresponding to phospho-sites significantly regulated in TRAP3^high^ cells (99 genes) compared to the set of genes identified across all proteomics analyses (9570 genes). Enriched GO terms are displayed by significance of the enrichment (q-value, multiple testing corrected) and fold enrichment. GO Molecular Function enrichment analysis (A). GO Cell Component enrichment analysis (B). (PDF 86 kb)

Additional file 7: Table S2. Proteins significantly regulated upon TRAP perturbation in any of the phosphoproteomics or proteomics analyses (TRAP3^high^ vs ctrl, phospho and standard proteomics analyses; TRAP sh2 or sh3 + 4 vs scrambled shRNA, standard proteomics analysis). The column “Enriched GO term” specifies whether the protein belong to any of the GO terms that were found to be enriched in the list of regulated proteins. The column “Protein class” describes to which of the examined protein class a protein belongs to. Examined proteins classes include protein kinases, protein phosphatases, ubiquitin and UBL system enzymes and transcription factors. (XLSX 79 kb)

Additional file 8: Figure S5. Functional analysis after treatment with TGFβ1 (10 μg/mL, A, B, *n* = 5) and respective controls. Live cell imaging was performed over 30 h in serum-free medium containing blocking antibody or small molecule inhibitor and wound migration velocity (A) and migration curves are compared. Cell proliferation was assessed after 24 h treatment in complete medium (B). Statistical comparison was performed on biological replicates by ANOVA test. Groups are generally compared to their respective controls (ctrl, untreated) or indicated with brackets and significance is annotated with an asterisk (*). ns = non-significant. “n=” indicates the number of replicates. (PDF 952 kb)

## Abbreviations

5-PNA: 5-phenylnicotinic acid
CD44: Cluster of differentiation 44
OPN: Osteopontin
TGFβ: Transforming growth factor β
TRAP/ACP5: Tartrate-resistant acid phosphatase
TβR: Transforming growth factor receptor β
