# Supplementary material for: Tartrate-resistant acid phosphatase (TRAP/ACP5) promotes metastasis-related properties via TGFβ2/TβR and CD44 in MDA-MB-231 breast cancer cells
Source: BMC Cancer. 2017 Sep 15;17:650. doi: 10.1186/s12885-017-3616-7 (PMC5602878; doi:10.1186/s12885-017-3616-7)
Supplement: Supplementary file 1 — Additional explanations on Material and Methods including descriptions of gene expression analysis, cell lysis, SILAC labeling, protein extraction for mass spectrometric analyses, protein digestion, Tandem Mass Tag labeling, liquid chromatography tandem mass spectrometric analyses and proteomics database search, protein and phospho-peptide ratios calculation. (DOCX 32 kb) [file 12885_2017_3616_MOESM1_ESM.docx]

**Additional Material and Methods**

## Gene expression Analysis

Total mRNA was extracted with the RNA-easy plus kit combined with QIAshredder columns (Qiagen, [Venlo](https://www.google.se/search?rlz=1C1BLWB_enSE567SE567&es_sm=93&q=venlo+netherlands&stick=H4sIAAAAAAAAAGOovnz8BQMDgzMHnxCnfq6-gaWxeZaFEgeIWZaVXaCllZ1spZ9flJ6Yl1mVWJKZn4fCscpITUwpLE0sKkktKvaomPbkG6cUn_Oq1ER1q6JPa51N5wMAenkjt2AAAAA&sa=X&ei=Wb39VJLEMIKdygPPrIGwCQ&ved=0CHwQmxMoATAP), [Limburg](https://www.google.se/search?rlz=1C1BLWB_enSE567SE567&es_sm=93&q=limburg+netherlands&stick=H4sIAAAAAAAAAGOovnz8BQMDgzMHnxCnfq6-gaWxeZaFEgeIaVJoYqSllZ1spZ9flJ6Yl1mVWJKZn4fCscpITUwpLE0sKkktKla4q3NCm_kql1fVTt3_Oyfmx_5ImA8AOKjiQWAAAAA&sa=X&ei=Wb39VJLEMIKdygPPrIGwCQ&ved=0CH0QmxMoAjAP), [Netherlands](https://www.google.se/search?rlz=1C1BLWB_enSE567SE567&es_sm=93&q=netherlands&stick=H4sIAAAAAAAAAGOovnz8BQMDgzMHnxCnfq6-gaWxeZaFEgeIaWqZZaSllZ1spZ9flJ6Yl1mVWJKZn4fCscpITUwpLE0sKkktKk55t2cFR83ZjU_sWBmuSnd-eugnaQoAAz6MjmAAAAA&sa=X&ei=Wb39VJLEMIKdygPPrIGwCQ&ved=0CH4QmxMoAzAP)) according to the manufacturer´s protocol and mRNA concentration quantified by spectrophotometry (Nanodrop 2000, Thermo Fisher Scientific, [Waltham](https://www.google.se/search?rlz=1C1BLWB_enSE567SE567&es_sm=93&q=waltham+ma&stick=H4sIAAAAAAAAAGOovnz8BQMDgwsHnxCXfq6-gVFBiYFJmhIHiJ1RZWyqpZWdbKWfX5SemJdZlViSmZ-HwrHKSE1MKSxNLCpJLSpOeVDm9ER_-4wmVYbdcam7J1UVy30CAGpinmJhAAAA&sa=X&ei=trv9VI-WDsPNygPf_oHoBA&ved=0CJABEJsTKAEwEA), [MA](https://www.google.se/search?rlz=1C1BLWB_enSE567SE567&es_sm=93&q=massachusetts&stick=H4sIAAAAAAAAAGOovnz8BQMDgysHnxCXfq6-gVFBiYFJmhIniG2abV6cpKWVnWyln1-UnpiXWZVYkpmfh8KxykhNTCksTSwqSS0q9qnfeleq9_H1E1f-1d6TXRQyMUfnGgCv4IEaYgAAAA&sa=X&ei=trv9VI-WDsPNygPf_oHoBA&ved=0CJEBEJsTKAIwEA), [U.S.](https://www.google.se/search?rlz=1C1BLWB_enSE567SE567&es_sm=93&q=united+states+of+america&stick=H4sIAAAAAAAAAGOovnz8BQMDgysHnxCXfq6-gVFBiYFJmhIniG2ZbF5uoKWVnWyln1-UnpiXWZVYkpmfh8KxykhNTCksTSwqSS0q1jnMy9Krc2G3Tf93hn07PerlnmR7AwCtSSfBYgAAAA&sa=X&ei=trv9VI-WDsPNygPf_oHoBA&ved=0CJIBEJsTKAMwEA)). 2 µg of mRNA were reversely transcribed into cDNA using Superscript III Reverse Transcriptase, oligo dT- Primers and dNTPs (Life technologies) according to the manufacturer´s protocol. Oligonucleotides were designed by Primer3 program (version 0.4.0) with sequences as follows. Human Actin: FW 5´-AAAGACCTGTACGCCAACACA-3´, RW 5´‑AGTACTTGCGCTCAGGAGGA-3´ (142 bp); Rat total TRAP: FW 5´-CGCCTACCTGTGTGGGCATGA-3´, RW 5´‑CACATAGCCCACACCGTTCTC-3´. Real- time gene amplification was performed according to the manufacturer´s protocol with the iQTM SYBR green Supermix (Biorad, [Hercules](https://www.google.se/search?rlz=1C1BLWB_enSE567SE567&es_sm=93&biw=1706&bih=1230&q=hercules+ca&stick=H4sIAAAAAAAAAGOovnz8BQMDgwsHnxCXfq6-gXGBYa55ihIHiF1YWViopZWdbKWfX5SemJdZlViSmZ-HwrHKSE1MKSxNLCpJLSre5Lb-_LvYIvO63qkrIqpMPXQfbNgMAK9MdyRhAAAA&sa=X&ei=fL39VMnbG4uBywOK1oKwDQ&ved=0CIgBEJsTKAEwEg), [CA](https://www.google.se/search?rlz=1C1BLWB_enSE567SE567&es_sm=93&biw=1706&bih=1230&q=ca&stick=H4sIAAAAAAAAAGOovnz8BQMDgwcHnxCXfq6-gXGBYa55ihKPfrq-oVFaelllmYmRllZ2spV-flF6Yl5mVWJJZn4eCscqIzUxpbA0sagktai4b13T5ddHn8XkMqb4rf20es7xH_b6AInbQEZlAAAA&sa=X&ei=fL39VMnbG4uBywOK1oKwDQ&ved=0CIkBEJsTKAIwEg), U.S.). Gene expression was normalized to β-Actin expression and quantified in the BioRad CFX manager 2.0 software.

## Cell Lysis

Protein extracts were prepared from cells grown for 24 h in complete medium (RPMI 1640 supplemented with 0.1 mg/mL Gentamicin and 10% fetal bovine serum) (Life technologies), respectively containing inhibitory compounds. For western blotting cell pellets were lysed in 50 µL cold RIPA-buffer (100 mM Tris-HCl pH 8, 300 mM NaCl, 2% NP40, 0.2 -2% SDS, 1% Sodium Deoxycholate) per 10^6^ cells. Extracts taken for enzyme activity assays were prepared in homogenization buffer (0.15 M KCl, 0.1% Triton X‑100) and 100 µL lysis buffer applied per 10^6^ cells. All lysates were freshly supplemented with complete protease inhibitor cocktail (Roche Diagnostics, Basel, Switzerland) and homogenized. Protein debris was removed and total protein content of lysates was determined by the use of Micro BCA Protein Assay Kit (Thermo Scientific) according to manufacturer´s descriptions. Respective conditioned media from the cells prepared for lysis, separated from cell debris and immediately processed at 4 ˚C.

## SILAC labeling

Stable isotope labeling with amino acids in cell culture (SILAC) was used for quantitative phosphoproteomics and standard proteomics analysis of control cells or TRAP3^high^ cells, as described before [1]. Briefly, control cells were grown in RPMI 1640 "Light medium", containing natural L-arginine-^12^C_6_, ^14^N_4_ (Sigma Cat# A6969) and L-lysine-^12^C_6_, ^14^N_2_ (Sigma Cat# L8662). TRAP3^high^ cells were grown in RPMI 1640 “Heavy medium” in which the natural arginine and lysine were replaced with heavy isotope-labeled amino acids L-arginine-^13^C_6_, ^15^N_4_ (Sigma Cat# 608033) and L-lysine-^13^C_6_, ^15^N_2_ (Sigma Cat# 608041). Cells were cultured for at least six doublings and labeling of more than 95% of the cellular proteins was confirmed by liquid chromatography tandem mass spectrometry (LC-MS) analysis before proceeding with the experiment.

## Protein extraction for mass spectrometric analyses

For SILAC-based phosphoproteomics and standard proteomics analysis of control cells and TRAP3^high^ cells, proteins were extracted at 50% cell confluence. Briefly, media was supplemented with 100nM Na_3_VO_4_ (100 nM) for 5 min at 37 °C to inhibit protein phosphatase activity, media was removed and culture dishes were then washed twice with ice-cold PBS. Cells were lysed by scraping in presence of 0.5% sodium deoxycholate (SDC), 0.35% sodium lauroyl sarcosinate (SLS), 50 mM HEPES pH 9.0 solution with 1 mM Na_3_VO_4_.

For Tandem Mass Tag (TMT)-based standard proteomics analysis of scrambled cells or TRAP knockdown cells, cell cultures around 70% confluence were harvested. Cell pellets were resuspended with 0.5% SDC, 0.35% SLS, 1 mM DTT, 50 mM HEPES pH 7.5 solution with Halt Protease and Phosphatase Inhibitor Cocktail (Thermo Fisher). Cell lysates were heated at 95 °C for 10 min, then sonicated and centrifuged for 15 min at 14 000 *g* and 4 °C. Protein concentration of the supernatants was measured using the DC-protein assay (Bio-Rad Laboratories, Hercules, CA, USA).

## Protein digestion

For SILAC-based phosphoproteomics and standard proteomics analysis of control and TRAP3^high^ cells, proteins were digested in solution. Briefly, cysteine residues were reduced with 1 mM dithiothreitol (DTT), lysates were sonicated, centrifuged for 15 min at 14 000 *g* and 4 °C. Protein concentration of the supernatants was measured using the DC-protein assay (Bio-Rad Laboratories, Hercules, CA, USA) and reduced cysteine residues were alkylated with 5.5 mM iodoacetamide (IAA). Finally, 2 mg of proteins per sample were digested at 37 °C o.n. with trypsin 1:80 w/w (Thermo Fisher) in the presence of 50 mM triethylammonium bicarbonate (TEAB) buffer pH 8.5 (Sigma-Aldrich). SDC was precipitated by adding trifluoroacetic acid (TFA) to 0.5% v/v (final concentration) and subsequently a phase separation was established using ethyl acetate, and the peptides were extracted from the aqueous phase [2]. Samples were desalted using Strong Cation Exchange-Solid Phase Extraction (SC-SPE) cartridges (Phenomenex) and peptide concentration was measured with DC protein assay (BioRad). Finally, peptides from control (Light SILAC labeled) and TRAP3^high^ (Heavy SILAC labeled) cells were mixed in a 1:1 proportion using 500 μg and 100 μg of peptides per sample for phosphoproteomics and standard proteomics analysis respectively. Samples were lyophilized in a speedvac and set aside for fractionation by high-resolution isoelectric focusing (HiRIEF).

For TMT-based standard proteomics analysis of scrambled or TRAP knockdown cells), proteins were digested following a slightly modified filter-aided sample preparation (FASP) protocol [3]. Briefly, 200 μg of proteins per sample were applied on 10k filtration units (Nanosep 10k Omega, Pall Life Sciences, Ann Arbor MI) and centrifuged. Cysteine residues were reduced using 1 mM DTT in 8 M urea-50 mM HEPES solution, followed by centrifugation and cysteine alkylation using 5.5 mM IAA in 4 M urea-50 mM solution. Filters were incubated for 10 min in the dark, centrifuged again and washed before digestion at 37 °C o.n. with trypsin 1:20 w/w (Thermo Fisher). Wash and digestion steps were performed using a 0.25 M urea-50 mM HEPES solution. The peptides were collected by centrifugation of the FASP filters, desalted using Strong Cation Exchange-Solid Phase Extraction (SC-SPE) cartridges (Phenomenex) and peptide concentration was measured with DC protein assay (BioRad). Aliquots containing 75 μg of peptides per sample were lyophilized in a speedvac and set aside for TMT labeling.

## Tandem Mass Tag labeling

Peptide samples from scrambled and knockdown cells (sh2 and sh3+4) were dissolved in 50 mM triethylammonium bicarbonate buffer (TEAB) pH 8.5. TMT10plex (Thermo Fisher) reagents were dissolved in 40 μl acetonitrile (ACN), added to each sample, and incubated for 3 h at R.T. with gentle shaking. The efficiency of labeling was determined by LC-MS prior to pooling samples. Pooled samples were desalted using Polymeric Reversed Phase-Solid Phase Extraction (RP-SPE) cartridges (Phenomenex), and then lyophilized in a speedvac prior to fractionation by high-resolution isoelectric focusing (HiRIEF).

## Liquid chromatography tandem mass spectrometric analyses

For phosphoproteomics analysis, the LC auto sampler (HPLC 1200 system, Agilent Technologies) dissolved each HiRIEF fraction in 8 μl of phase A (97% water, 3% ACN, 0.1% formic acid, FA) and injected 3 μl into a C18 guard desalting column (Zorbax 300SB-C18, 5x 0.3 mm, 5 μm bead size, Nikkyo Technos Co., Tokyo, Japan). Following 3 min of flow at 5 µl/min driven by the loading pump, the 6-port valve switched to analysis mode in which the nano gradient (NG) pump provided a flow of 250 nL/min through the guard desalting column. Chromatographic separation was performed on a 15 cm long C18 PicoFrit column (100 μm internal diameter, 5 μm bead size, Nikkyo Technos, Tokyo, Japan) installed on to the nano-electrospray ionization (NSI) source. The reversed phase gradient proceeded from an initial composition of 2% phase B (5% water, 95% ACN, 0.1% FA) to 40% phase B over 45 min, and finally to 100% phase B over the last 5 min. Upon completion of the gradient, the column was washed with a solution of 99% phase B for 10 min and re-equilibrated to the initial composition. Total LC-MS run time was 69 min.

MS analysis was performed using a hybrid LTQ Orbitrap Velos mass spectrometer (Thermo Scientific). Data-dependent MS/MS was performed on the 5 most abundant precursor ions from the master scans using collision-induced dissociation (CID) at 35% normalized collision energy followed by detection in the ion trap (ITMS). Precursor ions were isolated with a 4 m/z window and automated precursor ion dynamic exclusion was used with a 90 s duration. Precursor ions with unassigned charge state or a charge state of +1 were excluded. A precursor threshold of 1,000 counts was used. Automatic gain control (AGC) target number of ions was 1×10^6^ for MS1 and 2×10^4^ for CID-MS2. Maximum injection time for MS2 was 200 ms.

For standard proteomics analysis (both SILAC or TMT-based quantitative analysis), each HiRIEF fraction was dissolved in 15 µl of phase A (95% water, 5% dimethylsulfoxide (DMSO), 0.1% FA) by the auto sampler (Ultimate 3000 RSLC system, Thermo Scientific Dionex). For SILAC and TMT labeled peptides, 7 µl and 4 µl respectively were injected into a C18 guard desalting column (Acclaim pepmap 100, 75 µm x 2 cm, nanoViper, Thermo Scientific). Following 5 min of flow at 5 µl/min driven by the loading pump the 10-port valve switched to analysis mode in which the NG pump provided a flow of 250 nL/min through the guard desalting column. The curved gradient (specified as “curve 4” in Chromeleon software, Thermo Scientific) proceeded from an initial composition of 3% phase B (90% ACN, 5% DMSO, 5% water, 0.1% FA) to 45% phase B over 50 min. Upon completion of the gradient, the column was washed with a solution of 99% phase B and re-equilibrated to the initial composition. Total LC-MS run time was 74 min. A nano EASY-Spray column (pepmap RSLC, C18, 2 µm bead size, 100 Å, 75 µm internal diameter, 50 cm long, Thermo Scientific) was employed on the nano-electrospray ionization (NSI) EASY-Spray source (Thermo Scientific) at 60 °C. Online LC-MS was performed using a hybrid Q-Exactive mass spectrometer (Thermo Scientific). Fourier transform-based mass spectrometer (FTMS) master scans with a resolution of 70,000 (and mass range 300-1,600 m/z) were followed by data-dependent MS/MS (35,000 resolution) on 5 most abundant precursor ions using higher energy collision dissociation (HCD) at 30% normalized collision energy. Precursor ions were isolated with a 2 m/z window. Automatic gain control (AGC) target number of ions was 1×10^6^ for MS1 and 1×10^5^ for MS2. Maximum injection times were 100 ms for MS1 and 150 ms for MS2. The entire duty cycle lasted ~1.5 s. Automated precursor ion dynamic exclusion was used with a 60 s duration. Precursor ions with unassigned charge state or charge state of +1 were excluded. An underfill ratio of 1% was used.

## Proteomics database search

All tandem mass spectrometry (MS/MS) spectra were searched by Sequest/Percolator under the Proteome Discoverer software platform (PD 1.4, Thermo Scientific) using a target-decoy strategy. The reference database is the human protein subset of Swissprot, released 2016‑07‑08. A precursor ion mass tolerance of 10 ppm was used and product ion mass tolerances of 0.36 and 0.02 Da were used for CID-ITMS and HCD-FTMS respectively. Peptide spectral matches (PSMs) allowed for up to one missed trypsin cleavages (Lys-Pro and Arg-Pro were not considered cleavage sites).

To search MS/MS spectra generated by the SILAC-based quantitative analysis, carbamidomethylation on cysteine was set as fixed modification, whereas ^13^C_6_ ^15^N_4_ label on arginine, ^13^C_6_ ^15^N_2_ label on lysine and oxidation of methionine were set as dynamic modifications; additionally, phosphorylation of serine, threonine and tyrosine were included as dynamic modifications while searching MS/MS spectra from the phosphoproteomics analysis. The phosphoRS algorithm node was added to the workflow for the phosphoproteomics search to obtain probabilities of localization of phosphorylation sites [4], and only sites with high confidence of localization (≥ 75 pRS score) were used for quantification. To search MS/MS spectra generated by the TMT-based quantitative analysis, carbamidomethylation on cysteine and TMT-10 plex on lysine and N-terminus were set as static modifications and oxidation of methionine was set as dynamic modification. Quantitation of TMT-10 plex reporter ions was performed using an integration window tolerance of 10 ppm.

A false discovery rate (FDR) cutoff of 1% was applied at the peptide level for all searches.

## Protein and phospho-peptide ratios calculation

For SILAC-based phosphoproteomics and standard proteomics analysis of control and TRAP3^high^ cells, peptide-spectrum matches (PSMs) Heavy/Light (H/L) ratios were normalized to the median of all PSMs, assuming the majority of quantified peptides do not change abundance across experimental conditions. For phosphoproteomics analysis, phospho-site ratios were calculated as the median of all PSM ratios for a unique phospho-site. Phospho-sites are displayed as 15 amino acid sequences centered at the phosphorylated residue (sequence window). Analogously, protein ratios for SILAC-based standard proteomics analysis were calculated as the median over all PSM ratios for a unique gene. Phospho-site levels were normalized to changes in protein abundance by subtracting the log2 transformed protein ratio from the log2 transformed phospho-site ratio. For peptide count and plotting of the distribution across HiRIEF fractions, peptides were defined as unique by amino acidic sequence and number of phosphorylations. For each unique peptide, the HiRIEF fraction number of the PSM with the highest precursor area of all the PSMs corresponding to that peptide was taken as peptide fraction number.

For TMT-based standard proteomics analysis of scrambled cells or TRAP knockdown cells (sh2 and sh3+4), ratios were calculated first for all PSMs by dividing the intensity of each TMT channel by the average intensity of the three TMT channels corresponding to the scrambled samples. Protein ratios were calculated as the median over all PSM ratios for a unique gene. Furthermore, protein ratios were normalized to the median of all proteins per TMT channel, assuming equal protein loading of all 10 samples.

### References

1. Ong S-E, Mann M. A practical recipe for stable isotope labeling by amino acids in cell culture (SILAC). Nat. Protoc. [Internet]. Nature Publishing Group; 2007 [cited 2016 Dec 12];1:2650–60. Available from: http://www.nature.com/doifinder/10.1038/nprot.2006.427

2. Masuda T, Tomita M, Ishihama Y. Phase transfer surfactant-aided trypsin digestion for membrane proteome analysis. J. Proteome Res. 2008;7:731–40.

3. Wiśniewski JR, Zougman A, Nagaraj N, Mann M. Universal sample preparation method for proteome analysis. Nat. Methods [Internet]. 2009;6:359–62. Available from: http://www.ncbi.nlm.nih.gov/pubmed/19377485

4. Taus T, Köcher T, Pichler P, Paschke C, Schmidt A, Henrich C, et al. Universal and confident phosphorylation site localization using phosphoRS. J. Proteome Res. 2011;10:5354–62.
