# Supplementary figures and images for: Tartrate-resistant acid phosphatase (TRAP/ACP5) promotes metastasis-related properties via TGFβ2/TβR and CD44 in MDA-MB-231 breast cancer cells
Source: BMC Cancer. 2017 Sep 15;17:650. doi: 10.1186/s12885-017-3616-7 (PMC5602878; doi:10.1186/s12885-017-3616-7)

A

Replicate #1

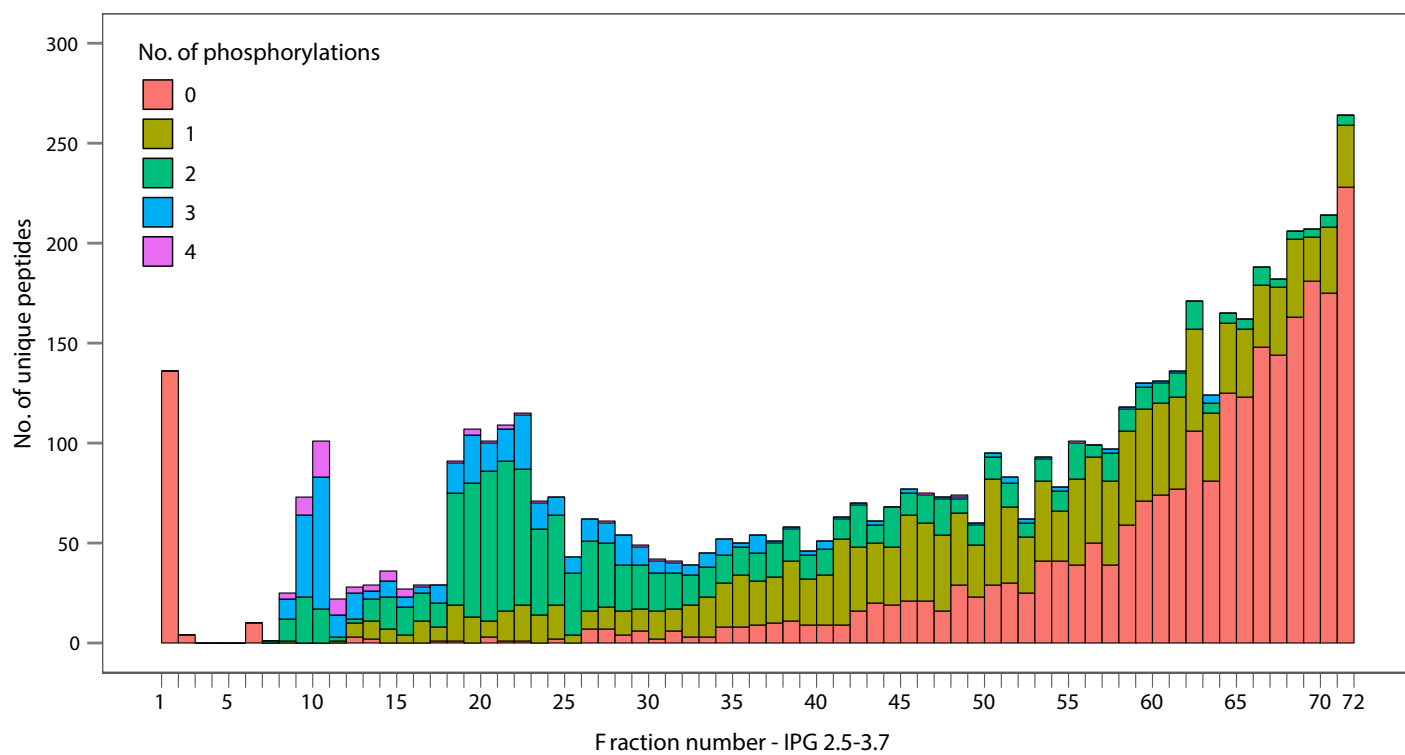

B

Replicate #2

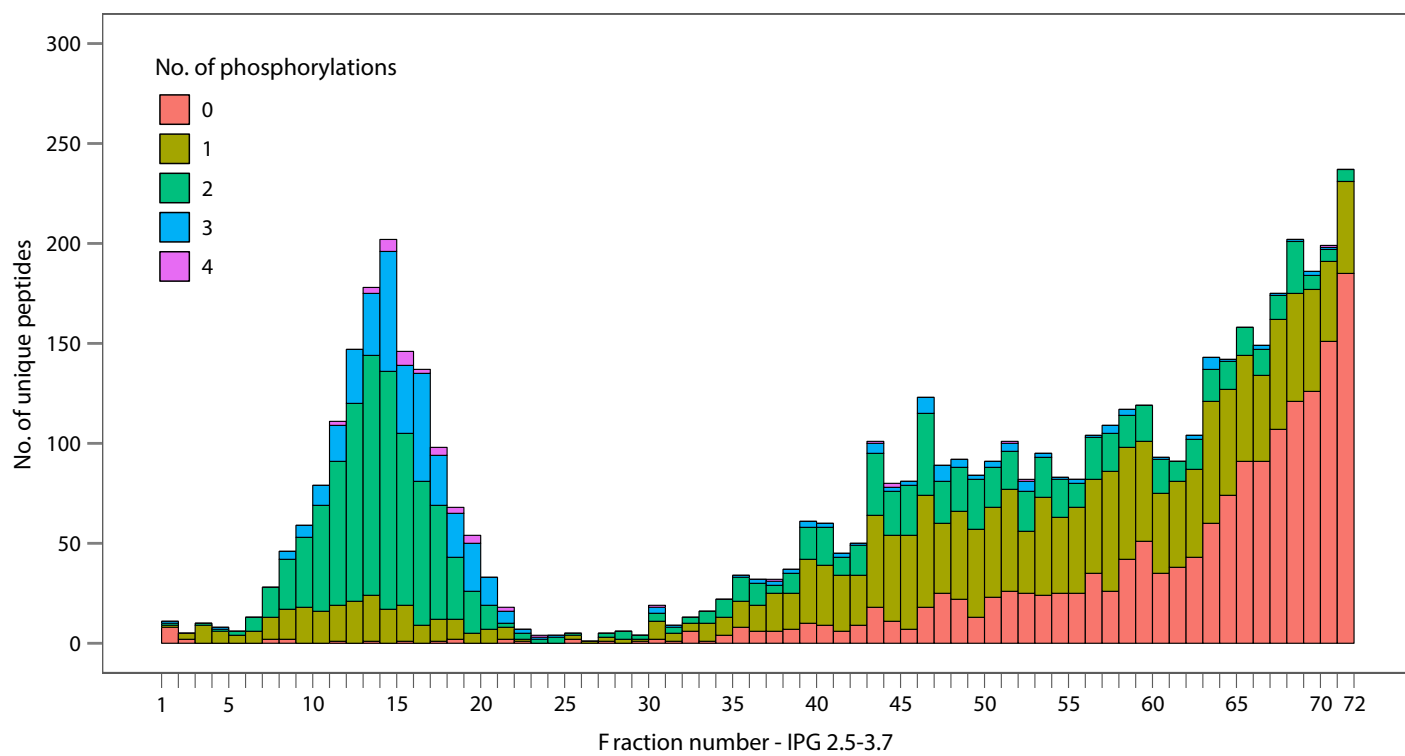

Supplement: Supplementary file 2 — Distribution of unique peptides across fractions generated by HiRIEF separation on IPG 2.5–3.7 strips. Number of unique peptides and phospho-peptides identified across fractions in each biological replicate (A) and (B), displayed by number of phosphorylations. Fraction numbering proceeds from the acidic end towards the basic end of the strips. Multiply phosphorylated peptides are identified prevalently in the first 30 fractions in the IPG 2.5–3.7 strip. (PDF 148 kb) [file 12885_2017_3616_MOESM2_ESM.pdf]

A

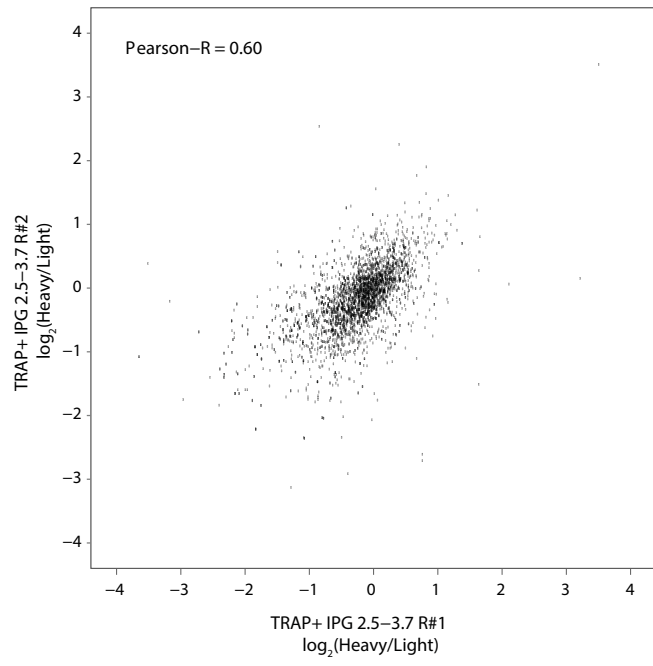

B

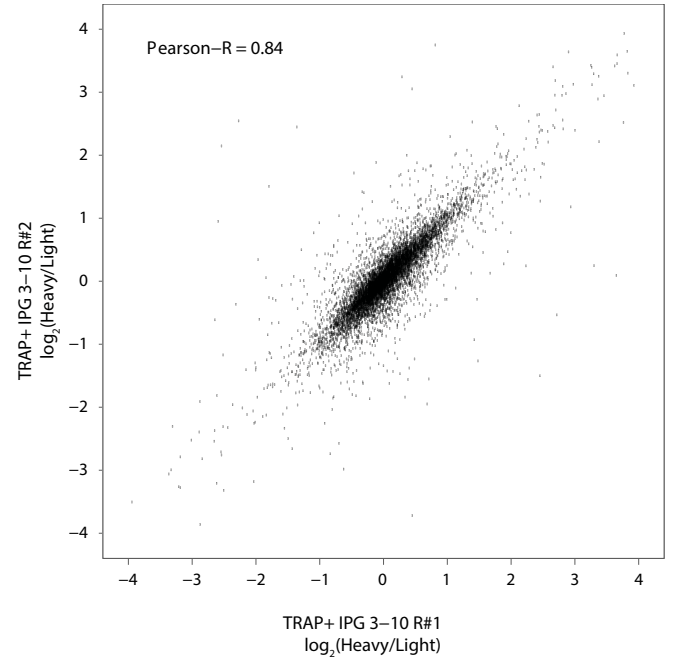

Supplement: Supplementary file 3 — Quantitative reproducibility of biological replicates employed for phosphoproteomics and proteomics analysis of TRAP3high (Heavy SILAC labeled) and control (Light SILAC labeled) MDA-MB-231 cells. Correlation of log2 transformed ratio (H/L) values for replicate pairs for phosphoproteomics analysis (A) and standard proteomics analysis (B); Pearson correlation coefficient is displayed. (PDF 2872 kb) [file 12885_2017_3616_MOESM3_ESM.pdf]

A

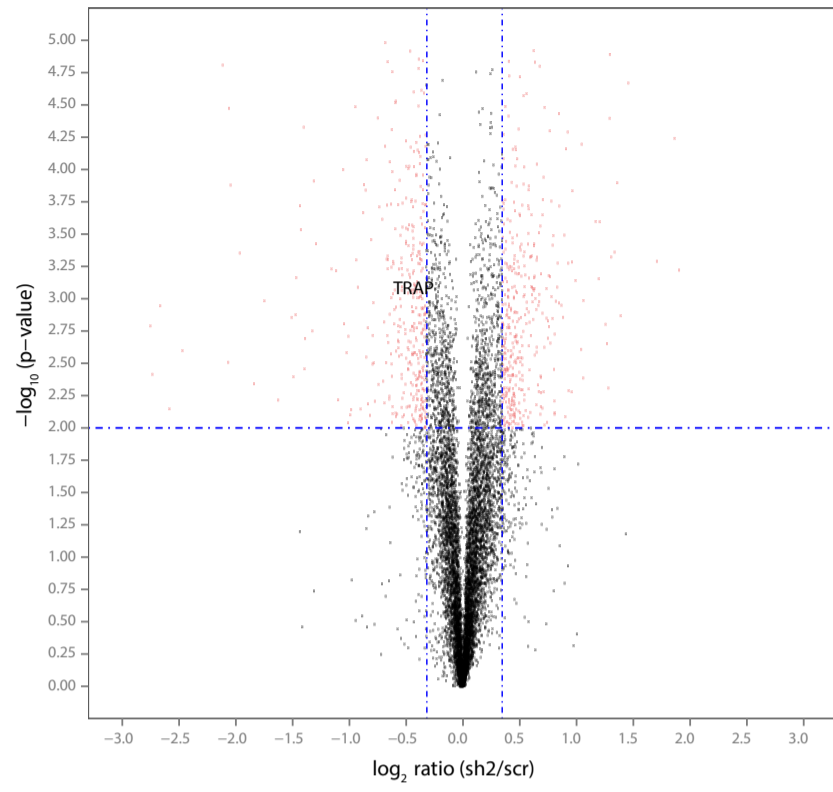

B

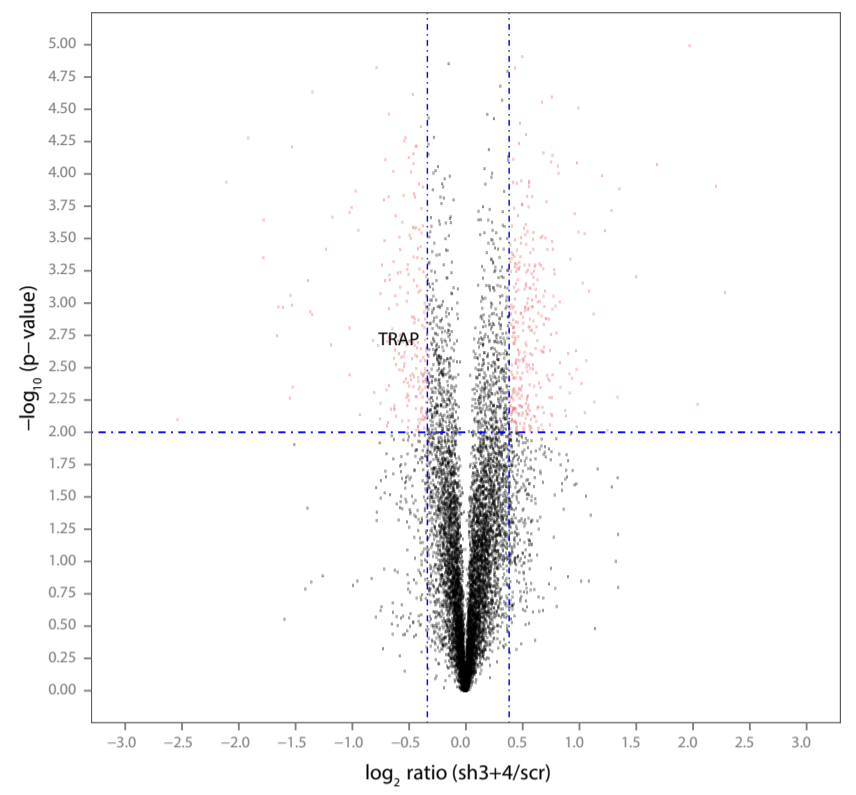

Supplement: Supplementary file 5 — Volcano plots representing the selection of proteins significantly regulated upon TRAP knockdown in TRAP3high MDA-MB-231 cells. The average log2 transformed ratio of the three replicates is plotted for each experimental condition. Significantly regulated events are defined by log2 transformed ratios at least −/+3 MAD away from the median and p-values lower than 0.01. TRAP sh2 samples versus scrambled (A). TRAP sh3 + 4 samples vs scrambled (B). (PDF 5171 kb) [file 12885_2017_3616_MOESM5_ESM.pdf]

A

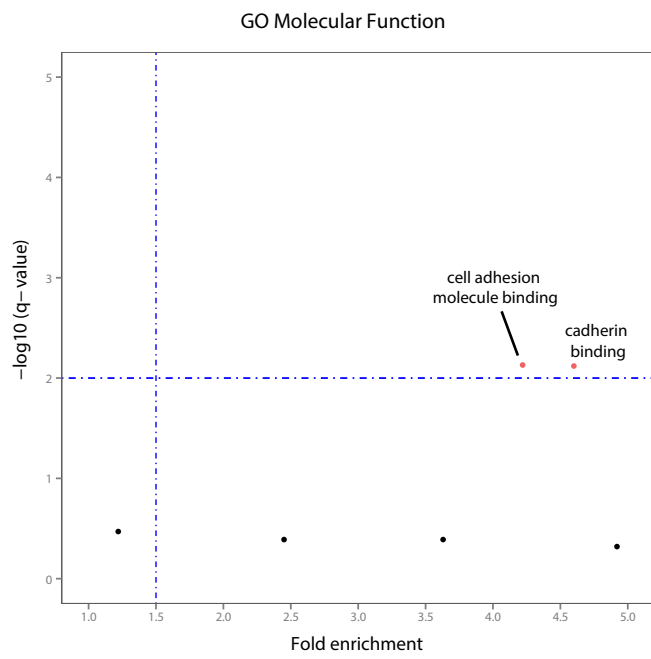

B

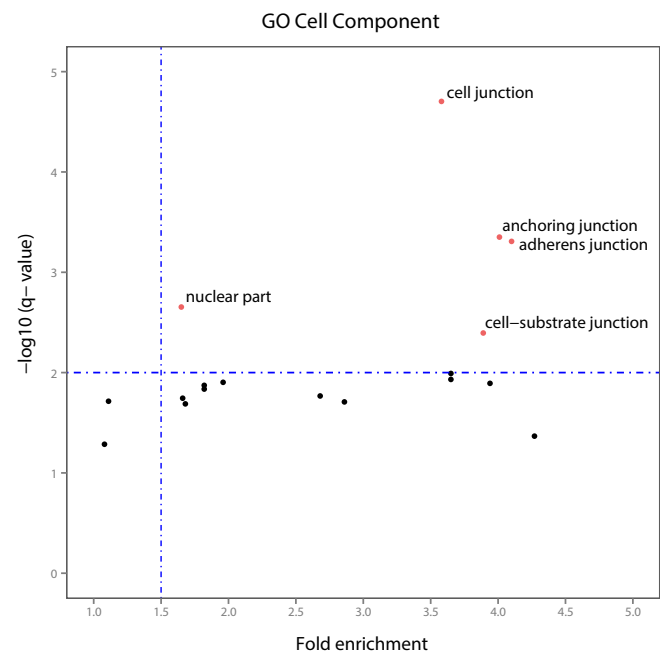

Supplement: Supplementary file 6 — GO enrichment analyses of genes corresponding to phosphorylation sites significantly regulated upon TRAP overexpression in MDA-MB-231 cells. GO terms enrichment was evaluated for the set of genes corresponding to phospho-sites significantly regulated in TRAP3high cells (99 genes) compared to the set of genes identified across all proteomics analyses (9570 genes). Enriched GO terms are displayed by significance of the enrichment (q-value, multiple testing corrected) and fold enrichment. GO Molecular Function enrichment analysis (A). GO Cell Component enrichment analysis (B). (PDF 86 kb) [file 12885_2017_3616_MOESM6_ESM.pdf]

A

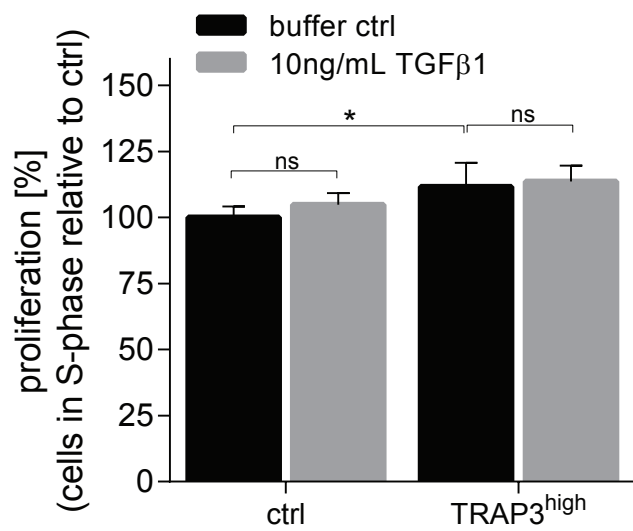

B

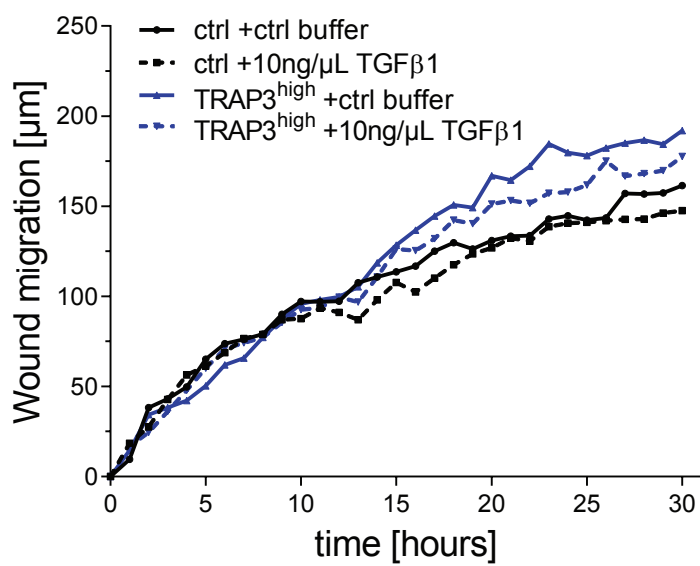

Supplement: Supplementary file 8 — Functional analysis after treatment with TGFβ1 (10 μg/mL, A, B, n = 5) and respective controls. Live cell imaging was performed over 30 h in serum-free medium containing blocking antibody or small molecule inhibitor and wound migration velocity (A) and migration curves are compared. Cell proliferation was assessed after 24 h treatment in complete medium (B). Statistical comparison was performed on biological replicates by ANOVA test. Groups are generally compared to their respective controls (ctrl, untreated) or indicated with brackets and significance is annotated with an asterisk (*). ns = non-significant. “n=” indicates the number of replicates. (PDF 952 kb) [file 12885_2017_3616_MOESM8_ESM.pdf]
